# Modifications in cellular viability, DNA damage and stress responses inflicted in cancer cells by copper-64 ions

**DOI:** 10.3389/fmed.2023.1197846

**Published:** 2023-06-21

**Authors:** Radu M. Serban, Dana Niculae, Gina Manda, Ionela Neagoe, Maria Dobre, Dragoș A. Niculae, Mihaela Temelie, Cosmin Mustăciosu, Radu A. Leonte, Livia E. Chilug, Maria R. Cornoiu, Diana Cocioabă, Miruna Stan, Anca Dinischiotu

**Affiliations:** ^1^Radiopharmaceutical Research Centre, Horia Hulubei National Institute for Physics and Nuclear Engineering (IFIN-HH), Măgurele, Ilfov, Romania; ^2^Faculty of Biology, University of Bucharest, Bucharest, Romania; ^3^Faculty of Pharmacy, University of Medicine and Pharmacy Carol Davila, Bucharest, Romania; ^4^Radiobiology Laboratory, National Institute of Pathology "Victor Babeș", Bucharest, Romania; ^5^Doctoral School of Applied Chemistry and Materials Science, Faculty of Chemical Engineering and Biotechnologies, University Politehnica of Bucharest, Bucharest, Romania; ^6^Doctoral School of Physics, Faculty of Physics, University of Bucharest, Măgurele, Ilfov, Romania

**Keywords:** auger-electrons, copper-64, radiotherapy, radiotoxicity, stress response, theragnostic

## Abstract

Due to combined therapeutical emissions, a high linear energy transfer Auger-electrons with the longer ranged β^−^ particles, ^64^Cu-based radiopharmaceuticals raise particular theragnostic interest in cancer, by joined therapeutic and real-time PET imaging properties. The *in vitro* study aimed to investigate the biological and molecular background of ^64^CuCl_2_ therapy by analyzing the damages and stress responses inflicted in various human normal and tumor cell lines. Colon (HT29 and HCT116) and prostate carcinoma (DU145) cell lines, as well as human normal BJ fibroblasts, were treated up to 72 h with 2–40 MBq/mL ^64^CuCl_2_. Radioisotope uptake and retention were assessed, and cell viability/death, DNA damage, oxidative stress, and the expression of 84 stress genes were investigated at various time points after [^64^Cu]CuCl_2_ addition. All the investigated cells incorporated ^64^Cu ions similarly, independent of their tumoral or normal status, but their fate after exposure to [^64^Cu]CuCl_2_ was cell-dependent. The most striking cytotoxic effects of the radioisotope were registered in colon carcinoma HCT116 cells, for which a substantial decrease in the number of metabolically active cells, and an increased DNA damage and oxidative stress were registered. The stress gene expression study highlighted the activation of both death and repair mechanisms in these cells, related to extrinsic apoptosis, necrosis/necroptosis or autophagy, and cell cycle arrest, nucleotide excision repair, antioxidant, and hypoxic responses, respectively. The *in vitro* study indicated that 40 MBq/mL [^64^Cu]CuCl_2_ delivers a therapeutic effect in human colon carcinoma, but its use is limited by harmful, yet lower effects on normal fibroblasts. The exposure of tumor cells to 20 MBq/mL [^64^Cu]CuCl_2_, might be used for a softer approach aiming for a lower radiotoxicity in normal fibroblasts as compared to tumor cells. This radioactive concentration was able to induce a persistent decrease in the number of metabolically active cells, accompanied by DNA damage and oxidative stress, associated with significant changes in stress gene expression in HCT116 colon cancer cells.

## 1. Introduction

The development of new therapeutic strategies using radioisotopes for oncologic pathologies is directed to ensure the delivery of as much as possible radiotoxicity and *in situ* damage to the targeted cancer cells but also to decrease the impact on healthy tissues. Given the importance of the copper ions in human metabolism, and their critical involvement in cancer growth and progression (1), copper radioisotopes emerged in recent studies to employ their properties for diagnostic and therapy follow-up by Positron Emission Tomography (PET) imaging (^61^Cu, ^64^Cu, ^62^Cu) (2–4) or their therapeutic potential (^67^Cu, ^64^Cu) (5, 6). Among copper radioisotopes of medical interest, ^64^Cu raises particular attention, as it decays either by electron capture (43.53%), positrons (β^+^) emission (17.52%), and by electrons (β^−^) emission (38.48%) (7). Therefore, ^64^Cu can be used for therapeutic purposes while PET images are acquired in real-time, being a promising theragnostic radiopharmaceutical. Still, its most promising feature lies in the cytotoxic effect of combined high linear energy transfer (LET) Auger-electrons with the lower LET but longer ranged electrons, β^−^. The Auger effect refers to the emission of a cascade of low-energy electrons co-produced by electron capture decay (Auger-K 6.54 keV, Auger-L 0.84 keV).

Understanding the mechanisms of copper interaction with tumor cells offers insights to improve therapeutic strategies (8, 9). Its relative biological effectiveness (RBE) is related to high LET radiation, a quality of α-particle emitters (80–100 keV/μm), and Auger electron emitters (4–26 keV/μm) translated in short penetration range of radiation, from tens of μm to less than 1 μm, respectively, (10, 11). While α-particles are considered densely ionizing radiation and β^−^-particles are sparsely ionizing, Auger-electrons form clusters with a high density of ionization events (12). Therefore, the utility of Auger-electrons emission is decisive for DNA targeting inside the cancer cell’s nucleus; however, recent studies suggest that the induction of cell death is possible if Auger electron emitters are in the cytoplasm or linked to a cell-membrane component, although the cytotoxic effect is less prominent (13, 14). Studies regarding ^125^I (also an Auger-electrons emitter) demonstrate that a significant contribution to RBE is due to oxidative stress-mediated nontargeted effects (13, 14). Other authors stipulate the involvement of indirect mechanisms, such as the crossfire effect of β^−^ decay and bystander effect, which occurs when neighboring cells in tissues communicate through gap junctions, and trigger cell death mechanisms with a larger magnitude than the phenomenon observed in cell-culture medium experiments (15, 16).

Copper is incorporated by human cancer cells through the human copper transporter-1 (hCTR1). For transport the ions need to be previously reduced by reductases from Cu^+2^ to Cu^+^, followed by selective permeation of Cu^+^ ions across the plasma membrane, bounding to metallochaperone proteins and delivery to the mitochondrion by the SLC25A3 inner membrane transporter. Copper is required for the metalation of cuproenzymes (ceruloplasmin, cytochrome C oxidase, metallothionein, Cu/Zn superoxide dismutase-1, amine oxidases, lysyl oxidase, tyrosinase, and mono-oxygenases) within the mitochondrial inter-membrane space (17–19). Elevated levels of copper promote the proliferation of various types of tumor cells (1, 19), such as colorectal cancer, breast cancer and lung cancer. Given the importance of genetic material replication, DNA damages and subsequent cell death are the main scope of radiation energy delivery in cancer. Biochemical mechanisms are those to establish if the genetic material is to be repaired or not (20). The disruptions in sensitive inner cell structures caused by ionization and oxidative stress can lead the death of tumor cells by apoptosis and autophagy (21).

The present study aimed to investigate *in vitro* relevant functional and molecular changes occurring in three cancer cell lines and one normal cell line after exposure to the emission of the theragnostic radioisotope ^64^Cu. We assessed modification of cellular viability and proliferation, DNA damage, oxidative stress, and how they correlate with the expression of relevant stress response genes.

## 2. Materials and methods

### 2.1. ^64^Cu production

^64^Cu production used enriched ^64^Ni electrodeposited on platinum support, attached to a shuttle target, in an automatic system (Alceo, Comecer, Italy) (22). Metallic ^64^Ni powder (99.53% enrichment) was solubilized in HNO_3_, and the pH was adjusted to 9.3 ± 0.02 with ammonium acetate/ammonium hydroxide. The resulting solution was transferred into the electrodeposition module, while the process was performed at a current intensity of 37 mA for 15–24 h to obtain a compact target, without stalagmites or cracks. The target was irradiated at the TR-19 cyclotron (ACSI, Richmond, Canada), *via* the ^64^Ni(p,n)^64^Cu nuclear reaction, using the following parameters: extracted beam energy 14.2 ± 0.3 MeV, energy on target 11.81 ± 0.63 MeV, integrated dose 150 μAh, according to an improved process of that previously described by our group (23, 24). After irradiation, the target was retracted and dissolved with 6 M HCl at 90°C. The resulting solution was automatically switched to the purification step, using an ion exchange resin (AG1X8) eluted with HCl solutions of different concentrations: 6 M HCl (recovering Ni), 4 M HCl (eluting Co impurities), and 0.5 M HCl collecting the ^64^CuCl_2_ (copper ions). The irradiation process yielded an activity between 10 and 16 GBq (decay-corrected to the end of bombardment). The solution pH was adjusted using NaOH to a physiological pH (7.2–7.4). The activity of the solution was measured using a VDC-405 dose calibrator (Veenstra Instruments, Netherlands), and the solution with the desired radioactive concentration was prepared using phosphate-buffered saline (PBS), pH 7.2–7.4 to adjust the final volume before incubation with the cell lines.

### 2.2. Cell cultivation

Three human tumors lines: HT29 (colon adenocarcinoma, ATCC HTB-38), HCT116 (colon carcinoma, ATCC CCL-247), DU145 (prostate carcinoma, ATCC HTB-81), and normal human skin fibroblast cell line BJ (ATCC CRL-2522) were cultivated according to the depositor’s proposed protocols (ATCC, American Tissue and Cell Collection) at 37°C and 5% CO_2_ in DMEM culture medium (Gibco, ThermoFisher Scientific, Waltham, MA, US) supplemented with 10% FBS (Euroclone, Milan, Italy), and a stabilized Antibiotic Antimycotic Solution (100x) (Sigma, Saint Louis, MO, US) containing 10,000 units penicillin, 10 mg streptomycin and 25 μg Amphotericin B per mL. This culture medium will be further designated as complete culture medium.

### 2.3. Incubation of cells with [^64^Cu]CuCl_2_

Different radioactive concentrations of the ^64^CuCl_2_ solution were tested (2 MBq/mL, 10 MBq/mL, 20 MBq/mL, and 40 MBq/mL). After 24 h of incubation of cells, allowing their adhesion (25), 10% of the cell culture medium was removed from cellular samples and the same volume of radioactive solution or complete culture medium was added to the test and control samples, respectively (26). For each sample, the ^64^Cu activity was adjusted before addition to cell culture, to obtain the desired radioactive concentration. Cells with and without ^64^CuCl_2_ solution were further incubated at 37°C in 5% CO_2_ atmosphere. The dynamics of functional and molecular changes inflicted by ^64^Cu radiotoxicity were investigated at various time points after the addition of the ^64^CuCl_2_ solution.

### 2.4. ^64^CuCl_2_ uptake and retention

^64^CuCl_2_ cellular uptake and retention was assessed with the LigandTracer Yellow equipment and the associated TraceDrawer software (LigandTracer 1.0.1 Ridgeview Instruments, Uppsala, Sweden), designed for the real-time monitoring of the interaction between a radionuclide or a radiolabelled compound with live cells. As previously described by our group (27), cells (400,000 cells/sample) were seeded in a defined section of a Petri dish of 91 mm diameter. The plate was maintained at an angle of 30° for 24 h to allow cells to adhere. Thereafter, plates were placed in the LigandTracer Yellow equipment which rotates the Petri dish and measures the radioactivity in 2 experimental settings: (i) cells in culture medium with radioisotope, and (ii) culture medium with radioisotope, without cells (28). Six points of measurement for each experimental setting were continuously measured and every 5.6 min a data point is recorded. The data point is calculated by the difference between the two settings to determine the radioactivity captured within cells. Results were presented as signal intensity (counts of radioactive decay per second) over time. The software compensates for the natural decay of the isotope. Radioisotope affinity for cell lines was analyzed by adding 18–24 MBq of [^64^Cu]CuCl_2_ solution to the culture medium. The retention of the radioisotope in the cell lines was analyzed by removing the medium, washing the cells with 1 mL of prewarmed complete culture medium, and adding 1 mL of fresh prewarmed complete culture medium.

### 2.5. Cell viability

For cellular viability analysis, 96-well plates were seeded with 5,000 fast-growing cells (DU145 and HCT116), and 10,000 slowly-growing cells (BJ and HT29) in a final volume of 100 μL. The MTS [3-(4,5-dimethylthiazol-2-yl)-5-(3-carboxymethoxyphenyl)-2-(4-sulfophenyl)-2H-tetrazolium, inner salt] reduction assay was used to evaluate the number of metabolically active cells in culture, based on the reduction of the MTS tetrazolium salt to formazan under the action of mitochondrial oxidoreductases. The MTS reduction test was performed with the CellTiter 96® AQueous One Solution Cell Proliferation Assay kit (Promega Corporation, Madison, WI, US), using the protocol recommended by the manufacturer (29). Briefly, at the end of the incubation time of cells with the ^64^Cu solution, 20 μL of the kit reagent was added to each triplicate sample. Cells were incubated for another 1 h at 37°C in 5% CO_2_ atmosphere, to allow the development of the MTS reduction reaction by metabolically active cells. Finally, the optical density (OD) of test and control samples was measured at 490 nm compared to the reference wavelength of 620 nm using the Tecan Sunrise reader and the Magellan data analysis program (Tecan Sunrise, Tecan, Männedorf, Switzerland). OD reading in each cellular test and control sample were corrected by subtracting the OD of the background samples containing only complete cell culture medium. Results were processed as mean OD value ± standard deviation of the mean (SEM) for triplicate samples.

### 2.6. Cell death

Plasma membrane integrity was evaluated by measuring the lactate dehydrogenase (LDH) released from cells following the damage in the plasma membrane of necrotic/necroptotic cells, using the CytoTox 96® Non-Radioactive Cytotoxicity Assay (Promega Corporation, Madison, WI, US) and the protocol provided by the producer (30). The experimental systems for LDH release were performed similarly to those of the MTS reduction test (point 2.5). At the end of the incubation time with the ^64^Cu solution, a volume of 50 μL of the cell culture supernatant was transferred to an empty well of a 96-well plate, where 50 μL of the kit reagent was added to each sample. Samples were then incubated for 30 min at room temperature in the dark to allow the LDH reaction to develop. The reaction was stopped with the special reagent in the kit. Finally, the optical density (OD) of test and control samples was measured at 490 nm compared to the reference wavelength of 620 nm using the Tecan Sunrise reader and the Magellan data analysis program (Tecan Sunrise, Tecan, Männedorf, Switzerland). OD reading in each cellular test and control sample were corrected by subtracting the OD of the background samples containing only complete cell culture medium. Results were processed as mean OD value ± standard deviation of the mean (SEM) for triplicate samples.

**Figure 1:**
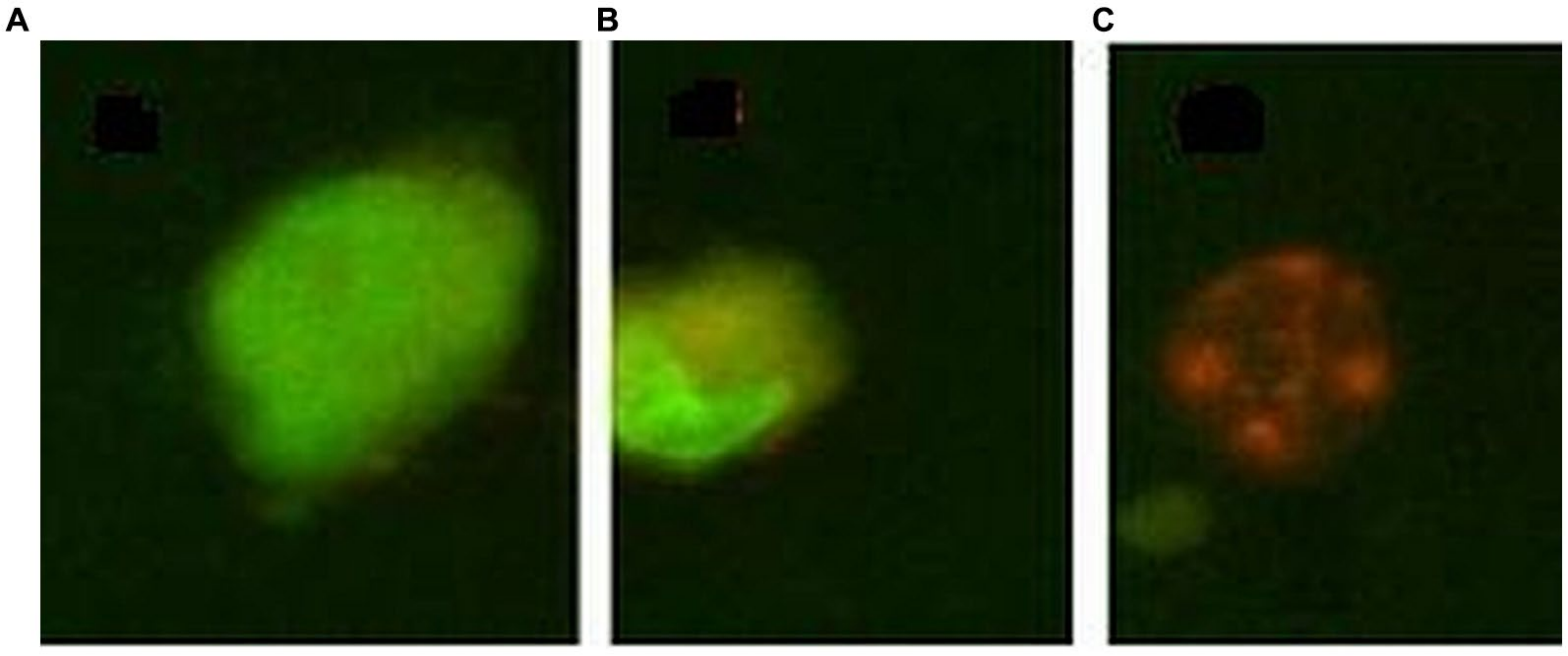
Exemple of morpholological staining of cells with acridine orange/ethidium homodimer 1: **(A)** – normal cell, **(B)** – apoptotic cell, **(C)** – necrotic cell.

The percentage of cells that died by apoptosis or necrosis was evaluated by morphological staining of cells with a mixture of acridine orange and ethidium homodimer-1. Acridine orange is a cell-permeant nucleic acid-selective fluorescent dye that emits green fluorescence (at 520 nm) when bound to double-stranded DNA and red fluorescence (at 650 nm) when bound to single-stranded DNA or RNA. Ethidium homodimer-1 is a cell-impermeant viability indicator, with a high affinity for nucleic acids that emits red fluorescence after binding to DNA in dead cells. Cells (50,000 cells/sample) were seeded in 24-well plates in a final volume of 1 mL culture medium. At the end of the incubation time with the [^64^Cu]CuCl_2_ solution, the supernatant of each sample was harvested and placed in Eppendorf tubes, and adhered cells were washed with 500 μL PBS at room temperature. The PBS solution used for cell washing was placed in the previously mentioned Eppendorf tubes for harvesting all detached cells from each sample. Adhered cells were detached from the wells with 300 μL of a 0.05%/0.02% (w/v) trypsin–EDTA solution (Biochrom AG, Berlin, Germany). The resulting cell suspension was added to the Eppendorf tube corresponding to each sample. The tubes were centrifuged at 400 g for 7 min, and the supernatant was discarded, leaving ~200 μL in each sample. Thereafter, 200 μL staining solution, containing 20 μg/mL acridine orange and 10 μg/mL ethidium homodimer in PBS, was added, and samples were further incubated in the dark for 20 min for labeling. Samples were centrifuged, the supernatant was discarded, and cells were washed in cold PBS by centrifugation to remove unbound stains. Cells were fixed for 15 min, in the dark with 1 mL 1% formaldehyde. The fixed cells were stored at 4°C in the dark until the radioactive dose decreased at the background level and samples could be transferred for fluorescent microscopy imaging. Briefly, samples were centrifuged, the supernatant was removed, and 20–30 μL of anti-fade mounting medium (2.3% DABCO in glycerol (Sigma-Aldrich CoSaint Louis, MO 63103, United States) was added to the cellular sediment. Eppendorf tubes were vortexed and transferred to glass slides for fluorescent microscopy analysis using a BX51 fluorescent microscope (Olympus, Tokyo, Japan). Green-colored cells with normal morphology were listed as living cells; cells with an orange cytoplasm or an abnormal morphology (nucleus condensation, cell tightening) were listed as apoptotic cells, while swollen or red-colored cells were listed as necrotic (Figure 1) (31).

### 2.7. Genotoxicity

The Comet assay method was used to evaluate the genotoxic effect of ^64^Cu. The experimental systems for genotoxicity were performed similarly to those for the MTS reduction test (point 2.5). Two control samples were prepared, a negative control consisting of an untreated sample and a positive control incubated for 30 min with 10 μM of hydrogen peroxide before analysis. After incubation in the presence or absence of the [^64^Cu]CuCl_2_ solution, cells were detached with 0.05%/0.02% (w/v) trypsin–EDTA solution (Biochrom AG, Berlin, Germany). A cell suspension from each sample was prepared in 0.5% low melting point agarose (LMPA) at 37°C and added to a microscope slide with 1% normal melting point agarose (NMPA) on ice. Slides were then immersed for 1 h at 4°C in an alkaline lysing solution (2.5 M NaCl, 100 mM EDTA, 10 mM Trizma base, 10% DMSO, 1% Triton X-100, pH 10). Slides were further immersed for 20 min in an electrophoresis tank filled with a 300 mM NaOH / 1 mM EDTA solution and were subjected to electrophoresis (15 V, 30 min, 300 mA). Slides were then washed three times for 5 min with a neutralizing solution (0.4 M Tris, pH 7.5). Cells were fixed for 5 min in a cold methanol bath (32). Slides were stored until the radioactive dose rate decreased at the background level. Before analysis, slides were hydrated for 15 min using 100 μL Vista Green staining solution (Cell Biolabs, INC, San Diego, CA, United States). Image acquisition and analysis were performed with the Olympus IX 71 fluorescence microscope (Tokyo, Japan), using the fluorescein isothiocyanate (FITC) filter, and the Comet Score™ software (TriTek Corporation, Sumerduck, VA, United States). The percentage of DNA present in the comet tail was considered a DNA damage indicator.

### 2.8. Redox status

The cellular redox status was evaluated as reduced glutathione (GSH) and malondialdehyde (MDA) content. Cells were seeded in 6 well plates (1 × 10^6^ cells/plate). After the treatment with the ^64^Cu solution, the supernatant was discarded, cells were detached with 0.05%/0.02% (w/v) trypsin–EDTA solution (Biochrom AG, Berlin, Germany), and were used for measuring the GSH and MDA levels.

The intracellular GSH levels were measured using the Glutathione Colorimetric Detection Kit from Sigma-Aldrich (Saint Louis, MO, US), according to the manufacturer’s indications (33). Cell pellets were washed in ice-cold PBS, were suspended at a ratio of 10^6^ cells/mL in ice-cold 5% 5-sulfo-salicylic acid (SSA), and were lysed by sonication on ice (3 times for 30 s each) with the Hielscher UP50H ultrasonic device (Hielscher Utrasonic, Teltow, Germany). After an incubation of 10 min at 4°C, samples were centrifuged at 14,000 rpm for 10 min at 4°C. The supernatant was collected and was diluted 20 times with the Assay Buffer and Sample Diluent (Assay Buffer for an initial 1:5 dilution then Sample Diluent was added for a final dilution of 1:20, as per manufacturer’s indications). In addition, serial dilutions of the standard were prepared, generating a total GSH concentration of 0, 0.781, 1.56, 3.125, 6.25, 12.5, and 25 μM. Standard dilutions and diluted samples (50 μL each) were placed in a 96-well plate and were mixed with 25 μL of Colorimetric Detection Reagent and with 25 μL of Reaction Mixture. After an incubation of 20 min at room temperature, the absorbance was read at 405 nm at an ELISA reader (FlexStation 3, Molecular Devices, Sunnyvale, CA, United States). The GSH concentration was determined from the standard curve plot and was adjusted with the dilution factor.

Lipid peroxidation as a result of oxidative stress was evaluated by measuring MDA levels using the Lipid Peroxidation (MDA) Assay Kit from Sigma-Aldrich (Saint Louis, MO, US), according to the manufacturer’s indications (34). Briefly, MDA standard dilutions (0, 0.4, 0.8, 1.6, and 2.0 nmoles) were prepared. Cell pellets (~10^6^ cells) were homogenized on ice in 300 μL MDA lysis buffer containing 3 μL of butylated hydroxytoluene (BHT). The insoluble material was removed by centrifugation at 13,000 × g for 10 min. The supernatant from each homogenized sample (200 μL) was placed into a microcentrifuge tube, was incubated for 60 min at 95°C with 600 μL thiobarbituric acid (TBA) solution, and was then chilled to room temperature in an ice bath for 10 min. Finally, 200 μL from each reaction mixture was placed into a black 96-well plate with clear bottom, and the fluorescence intensity was measured at an FP-750 spectrofluorometer (Jasco, Tokyo, Japan), using λ_excitation_ = 532 nm and λ_emission_ = 553 nm. The background was corrected by subtracting the blank value from all readings. The amount of MDA present in the samples was determined from the standard curve plot and was adjusted with the sample dilution factor.

### 2.9. Gene expression

The expression level of 84 stress genes (Supplementary File 1; Table 1) addressing cell death, DNA damage, and repair, unfolded protein response, as well as oxidative, hypoxic, osmotic, and inflammatory stress, was investigated by qRT-PCR. Briefly, cells were seeded in 6 well plates (1 × 10^6^ cells/well), were allowed to adhere for 24 h, and were thereafter treated with the [^64^Cu]CuCl_2_ solution (20 MBq/mL) for another 24 h. The supernatant was discarded and adherent cells were washed twice with PBS at room temperature. Cells were detached with 0.05%/0.02% (w/v) trypsin–EDTA solution (Biochrom AG, Berlin, Germany), were washed twice by centrifugation in cold PBS, and were finally treated with 1 mL RiboZol RNA Extraction Reagent (Avantor, Bridgeport, NJ, US). Samples were stored at −80°C in RiboZol, at least until the radioactive dose rate decreased at the background level. For investigating the expression of stress genes, total RNA was isolated from cells preserved in RiboZol according to the manufacturer’s instructions, and RNA concentration was assessed with the Nanodrop 2000 equipment (Thermo Fisher Scientific, Waltham, MA, US). Both the 260/280 nm and 260/230 nm ratios were > 1.8. cDNA synthesis was performed using ~600 ng of total RNA with the RT^2^ First Strand Kit (Qiagen, Hilden, Germany), according to the manufacturer’s instructions. The expression of the stress genes was assessed using the RT2 Profiler™PCR Array Human Stress and Toxicity PathwayFinder (Qiagen, PAHS-003Z), using the SYBR Green chemistry on an ABI7500 Fast PCR System (Thermo Fisher Scientific, Waltham, MA, US). The expression level of each gene was normalized with the geometric mean value of one housekeeping genes (GAPDH) that was selected based on its stability along all the experimental settings using the online RefFinder algorithm (35) from the five candidate housekeeping genes contained in the kit (ACTB, B2M, GAPDH, HPRT1, and RPLP0). Gene expression data were analyzed with the RT^2^ Profiler PCR Array software package (Qiagen, Hilden, Germany). Gene expression levels were calculated as 2^-ΔCT^ values. The Fold Change value (FC) was calculated as the 2^-ΔCT^ value in the cellular sample treated with the ^64^Cu solution divided by the 2^-ΔCT^ value in the non-treated control. FC > 1.5 indicates gene transcription upregulation, while FC < 0.7 downregulation.

**Table 1:** The 84 stress genes analyzed.

DNA damage & repair		
*Cell Cycle Arrest & Checkpoints*: CDKN1A (p21CIP1, WAF1), CHEK1, CHEK2 (RAD53), DDIT3 (GADD153, CHOP), HUS1, MRE11, NBN, RAD17, RAD9A	*Other DNA Damage Responses*: ATM, ATR, DDB2, GADD45A, GADD45G, RAD51, TP53 (p53), XPC.	
*Unfolded Protein Response*: ATF4, ATF6, ATF6B, BBC3 (PUMA), BID, CALR, DDIT3 (GADD153, CHOP), DNAJC3, HSP90AA1, HSP90B1, HSPA4 (HSP70), HSPA5 (GRP78).		
Cell death		
*Apoptosis*: CASP1 (ICE), FAS, MCL1, TNFRSF10A (TRAIL-R), TNFRSF10B (DR5), TNFRSF1A (TNFR1)	*Necrosis*: FAS, GRB2, PARP1 (ADPRT1), PVR, RIPK1, TNFRSF10A (TRAIL-R)	*Autophagy*: ATG12, ATG5, ATG7, BECN1, FAS, ULK1
OXIDATIVE STRESS: FTH1, GCLC, GCLM, GSR, GSTP1, HMOX1, NQO1, PRDX1, SQSTM1, TXN, TXNRD1	HIPOXIA SIGNALING: ADM, ARNT, BNIP3L, CA9, EPO, HMOX1, LDHA, MMP9, SERPINE1 (PAI-1), SLC2A1, VEGFA	
OSMOTIC STRESS: AKR1B1, AQP1, AQP2, AQP4, CFTR, EDN1, HSPA4L (OSP94), NFAT5, SLC5A3	INFLAMMATORY RESPONSE: CCL2 (MCP-1), CD40LG, CRP, CXCL8 (IL8), IFNG, IL1A, IL1B, IL6, TLR4, TNF	

### 2.10. Statistical analysis of data

Whenever possible, data were presented as mean value ± standard error of the mean (SEM) for triplicate samples. Comparison between triplicate samples treated with the [^64^Cu]CuCl_2_ solution and triplicate untreated controls was performed with the Student’s t-test. For the comet assay, due to data dispersion, we selected the P_25_-P_75_ interval for statistical analysis. Given the fact that the comet assay data were not normally distributed according to the Shapiro–Wilk test, the Mann–Whitney U test was used to compare samples treated with ^64^CuCl_2_ or H_2_O_2_ and untreated controls. Differences between treated samples and controls were considered significant for *p* values <0.05. The half maximal effective activity (EC_50_) was calculated using the online calculator freely provided by AAT Bioquest (36).

## 3. Results

### 3.1. ^64^Cu uptake and retention in normal and tumor cell lines

**Figure 2:**
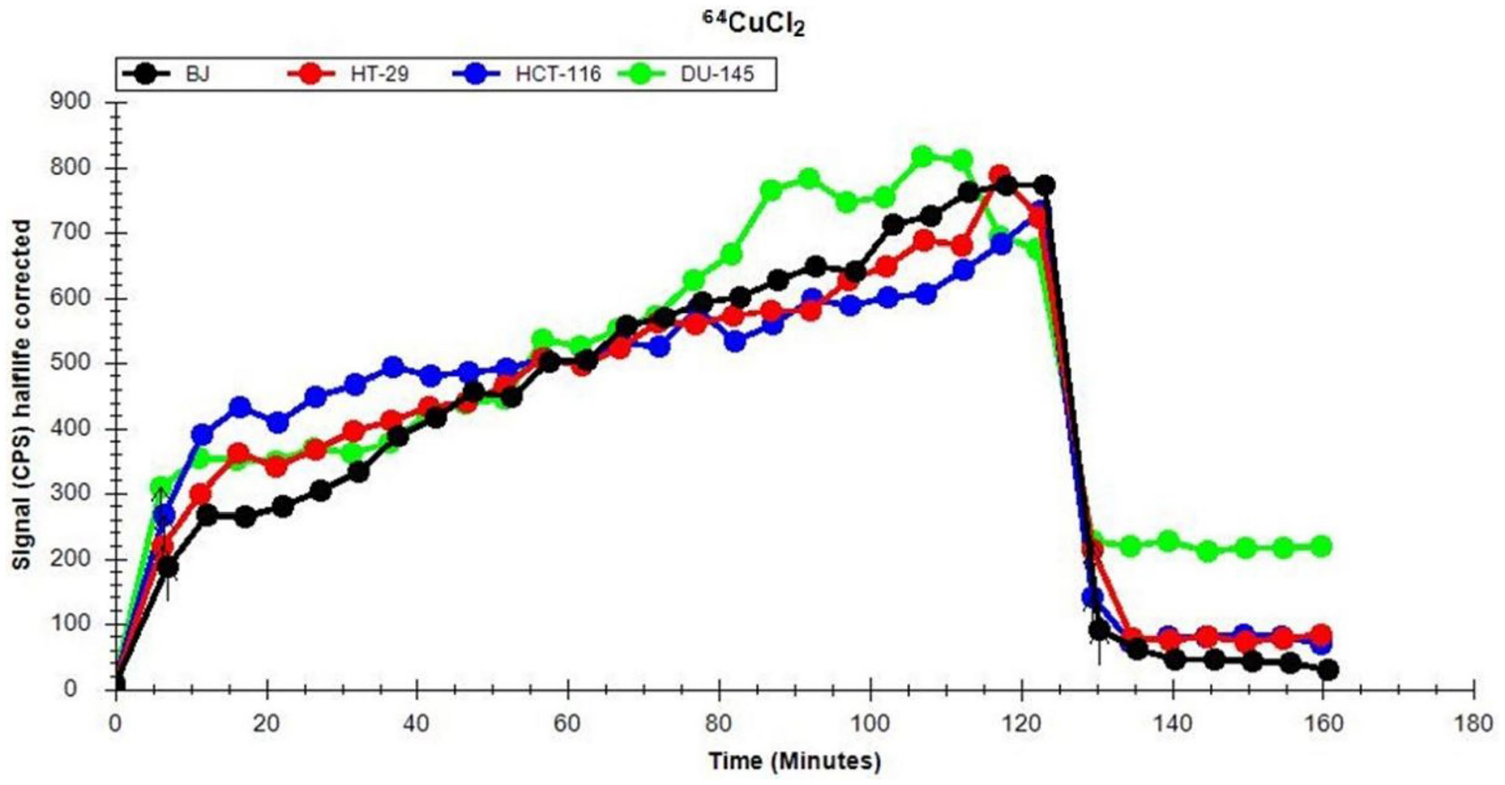
Representative dynamics of ^64^Cu uptake and retention of (21 ± 3 MBq ^64^Cu) in human normal and tumor cells lines, evaluated using the LigandTracer method.

The uptake and retention of ^64^Cu ions in human normal (BJ fibroblasts) and tumor cell lines (colon HT29 and HCT116 cells or prostate carcinoma DU145 cells) was investigated using the LigandTracer method (27, 37). Data (Figure 2) show that, after a 2 h incubation of cells with the ^64^CuCl_2_ solution (21 ± 3 MBq), all the investigated cell lines presented comparable ^64^Cu uptake at 120 min after the addition of the radioactive solution.

Elimination of ^64^Cu from the extracellular environment resulted within 10 min in an abrupt decrease of the incorporated radioactivity to less than 30% (Figure 2). This is the reason why, for analyzing long-term cellular effects of the radioisotope, cells were continuously exposed to the[^64^Cu]CuCl_2_ solution for 24 h, 48 h, and 72 h, when only the radioactivity decay should be taken into consideration (T_1/2_ = 12.7 h).

After the washout of the radioactive solution, a rapid stabilization between the radioactivity retained within cells and the radioactivity released by cells in the culture medium was observed (Figure 2). Statistical analysis of the data corresponding to the retention part of the graphic (Figure 2) was performed on 7 independent experiments. Results indicate that there are no significant differences between the investigated cell lines regarding the retention of ^64^Cu ions. As such, distinctive cellular effects of ^64^CuCl_2_ may not be due to differences in radioisotope uptake and/or retention, but to cell type-dependent stress response mechanisms (38), as we will describe in the following chapters.

### 3.2. The effects of ^64^Cu on the number of metabolically active cells

The timely effect exerted by ^64^CuCl_2_ on the MTS reduction by human normal BJ fibroblasts and several human tumor cells (colon carcinoma HT29 and HCT116 cells and prostate carcinoma DU145 cells) was investigated for assessing the effects on the number of metabolically active cells in treated and non-treated cultures. The ^64^CuCl_2_ effect was assessed in the dose range of 2–40 MBq/mL, which encompasses both radioactive doses used for PET images (2 MBq/mL) (39, 40) and higher, potentially therapeutic doses (10, 20, and 40 MBq/mL). Cells were monitored at 24 h, 48 h, and 72 h after the addition of the ^64^CuCl_2_ solution to cell cultures. Based on the data obtained for radioisotope uptake and retention (Figure 2), cells were continuously exposed to the ^64^CuCl_2_ solution, but the decay of ^64^Cu radioactivity in the time frame of cellular investigations should be taken into consideration (Supplementary File 1; Figure 1).

**Figure 3:**
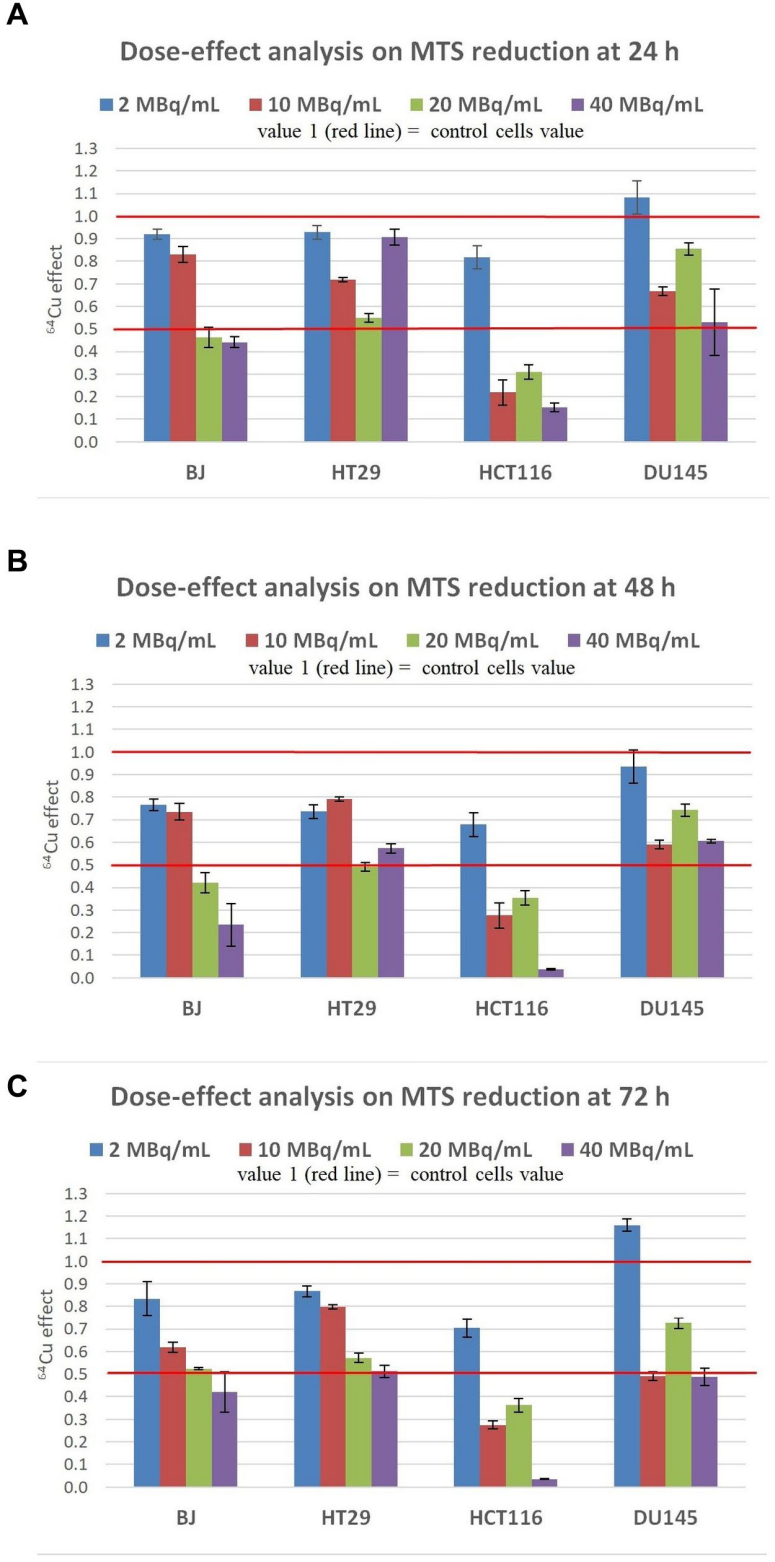
Dose-effect analysis on MTS reduction by human normal BJ fibroblasts and by various human tumor cells (HT29 and HCT116 colon carcinoma, and DU145 prostate carcinoma) exposed to ^64^Cu in the dose range of 2, 10, 20 and 40 MBq/mL for various time points: **(A)** 24 h; **(B)** 48 h; **(C)** 72 h. Results are presented as mean ± SEM for triplicate samples. ^64^Cu effect was calculated as MTS reduction value (OD) for each sample of the triplicate divided by the mean value of MTS reduction in the non-treated controls.

At 24 h post-exposure of cells to 2 MBq/mL ^64^Cu (Figure 3A), the lowest radioactive concentration investigated, induced only a relatively minor decrease of MTS reduction by normal BJ and colon carcinoma HT29 cells, and no significant changes in prostate carcinoma DU145cells. Meanwhile, colon carcinoma HCT116 cells were more affected by ^64^Cu radioactive emissions than the other investigated cell lines, exhibiting around a 15% decrease in the MTS reduction as compared to untreated cells. By increasing the ^64^Cu dose to 10 MBq/mL, a significant decrease of MTS reduction was registered at 24 h in the case of HCT116, HT29, and DU145 cells, with the most pronounced effects being displayed in human colon carcinoma HCT116 cells for which MTS reduction decreased to 20% of the untreated cells value. As such, these tumor cells seem to have an early reaction to 10 MBq/mL, observed at 24 h, unlike normal BJ fibroblasts that almost did not sense the dose increase from 2 MBq/mL to 10 MBq/mL. An even more significant decrease of MTS reduction was induced at 24 h by 20 MBq/mL ^64^Cu in all the investigated cell lines, with the most pronounced effects being again observed for colon carcinoma HCT116 cells for which MTS reduction decreased to ~30% as compared to untreated cells. Of note is that these cells reacted almost similarly to the lower radioactive concentration of 10 MBq/mL, and the increase to 20 MBq/mL did not bring any additional decrease in MTS reduction intensity. A further increase to 40 MBq/mL did not induce a dose-dependent response in BJ normal cells, or in HT29 and DU145 tumor cells, the MTS reduction level remaining almost at the value noticed when cells were treated with the radioactive concentration of 20 MBq/mL ^64^Cu. In the case of the highly reactive HCT116 cells, there was a slight decrease in MTS reduction at 40 MBq/mL as compared to 20 MBq/mL.

**Figure 4:**
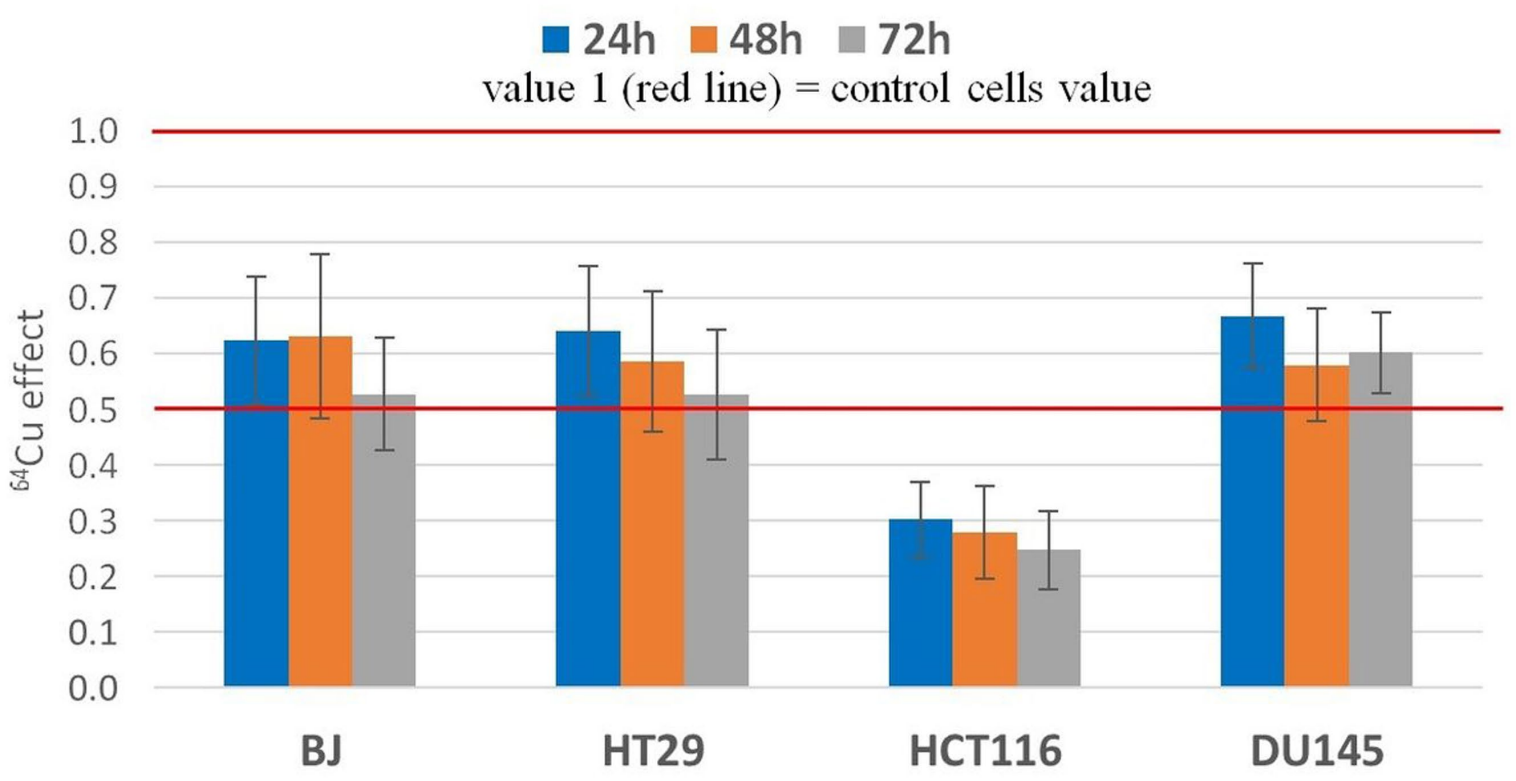
Statistic effect of 20 MBq/mL ^64^Cu on the MTS reduction by normal human BJ fibroblasts and by various human tumor cells (HT29 and HCT116 colon carcinoma and DU145) prostate carcinoma cells. Results are presented as mean value ± SEM for *n* = 3 independent experiments. The mean effect was calculated in each experiment as parameter value in exposed cells divided by the parameter value in non-exposed cells (controls), and was further used for the statistics of 3 independent experiments.

The trend of ^64^Cu effects on the investigated tumor and normal cell lines registered at 24 h (Figure 3A) was maintained at 48 h after the addition of the [^64^Cu]CuCl_2_ solution. Results indicate persistent effects of ^64^Cu, albeit the decay of ^64^Cu in this time interval. An additional decrease of MTS reduction was induced by the exposure of BJ, HT29, and HCT116 cells to the low radioactive concentration of 2 MBq/mL at 48 h as compared to 24 h. Similar decreases in MTS reduction were registered at 48 h in BJ and HT29 cells exposed to 2 MBq/mL and 10 MBq/mL, indicating that these cells are not affected by the slightly increased radioactive concentration. Meanwhile, all cell lines reacted starting early, at 24 h, to a radioactive concentration increased to 20 MBq/mL (Figure 3A), when an important decrease of MTS reduction was registered, and the effect was persistent also at 48 h (Figure 3B). A further radioactive concentration increase to 40 MBq/mL did not bring additional changes of MTS reduction at 48 h (Figure 3B) as compared to 24 h (Figure 4A), except for HCT116 cells for which MTS reduction decreased to ~10% of the value registered in untreated cells. In presence of 40 MBq/mL [^64^Cu]CuCl_2_ most of the HCT116 cells died, as microscopic evaluation showed that most of the cells were detached (data not shown).

The inhibitory effects exerted by ^64^Cu on MTS reduction at 48 h (Figure 3B) continued to be stable at 72 h after the exposure of cells to ^64^CuCl_2_ (Figure 3C). Only in the case of normal BJ fibroblasts, an additional decrease of MTS reduction triggered by 10 MBq/mL [^64^Cu]CuCl_2_ was observed at 72 h, resulting in a clear dose-effect relationship at 72 h after radioisotope addition (y = −0.0183 + 0.8456; R^2^ = 0.9497).

Altogether, results indicate that the inhibitory effects of ^64^Cu on MTS reduction and, consequently, on the number of metabolically active cells in culture, were exerted in the first 24 h after cells exposure to the radiation emitted by copper-64, with the most pronounced decrease of MTS reduction being registered in HCT116 colon carcinoma cells. This effect was maintained for 72 h after ^64^Cu addition. EC_50_ calculations at 72 h ordered the reactivity of the investigated cells to ^64^Cu as follows: human normal BJ fibroblasts (EC_50_ = 12.63) ≤ human HT29 colon adenocarcinoma (EC_50_ = 12.46) < human DU145 prostate carcinoma (EC_50_ = 9.46) < human HCT116 colon carcinoma (EC_50_ = 5.63). Of note is that not only tumor cells but also normal BJ fibroblasts were affected by the exposure to ^64^Cu, even at the lower dose of 2 MBq/mL (39).

The effects exerted by 20 MBq/mL [^64^Cu]CuCl_2_ on MTS reduction by normal and tumor cells were validated in 3 independent experiments (Figure 4). The statistical analysis confirmed the previously presented results (Figure 3), showing that the most noticeable decrease of MTS reduction triggered by ^64^Cu was registered in HCT116 colon carcinoma cells, as compared to the other investigated cell lines (*p* < 0.001), for which MTS reduction was decreased to ~30% of the untreated cells. Meanwhile, the ^64^Cu treatment decreased up to 50% the MTS reduction by normal BJ fibroblasts and colon carcinoma HT29 cells, and only to about 60–70% by prostate carcinoma DU145 cells. Statistically, similar effects of ^64^CuCl_2_ were registered at 24 h, 48 h, and 72 h after radioisotope addition (Figure 4) for each of the investigated cell lines, indicating that ^64^Cu exerted its radiotoxic effects within the first 24 h of exposure, and these effects were persistent, being maintained at least for a 72 h incubation time.

### 3.3. The genotoxic effect of the ^64^Cu

**Figure 5:**
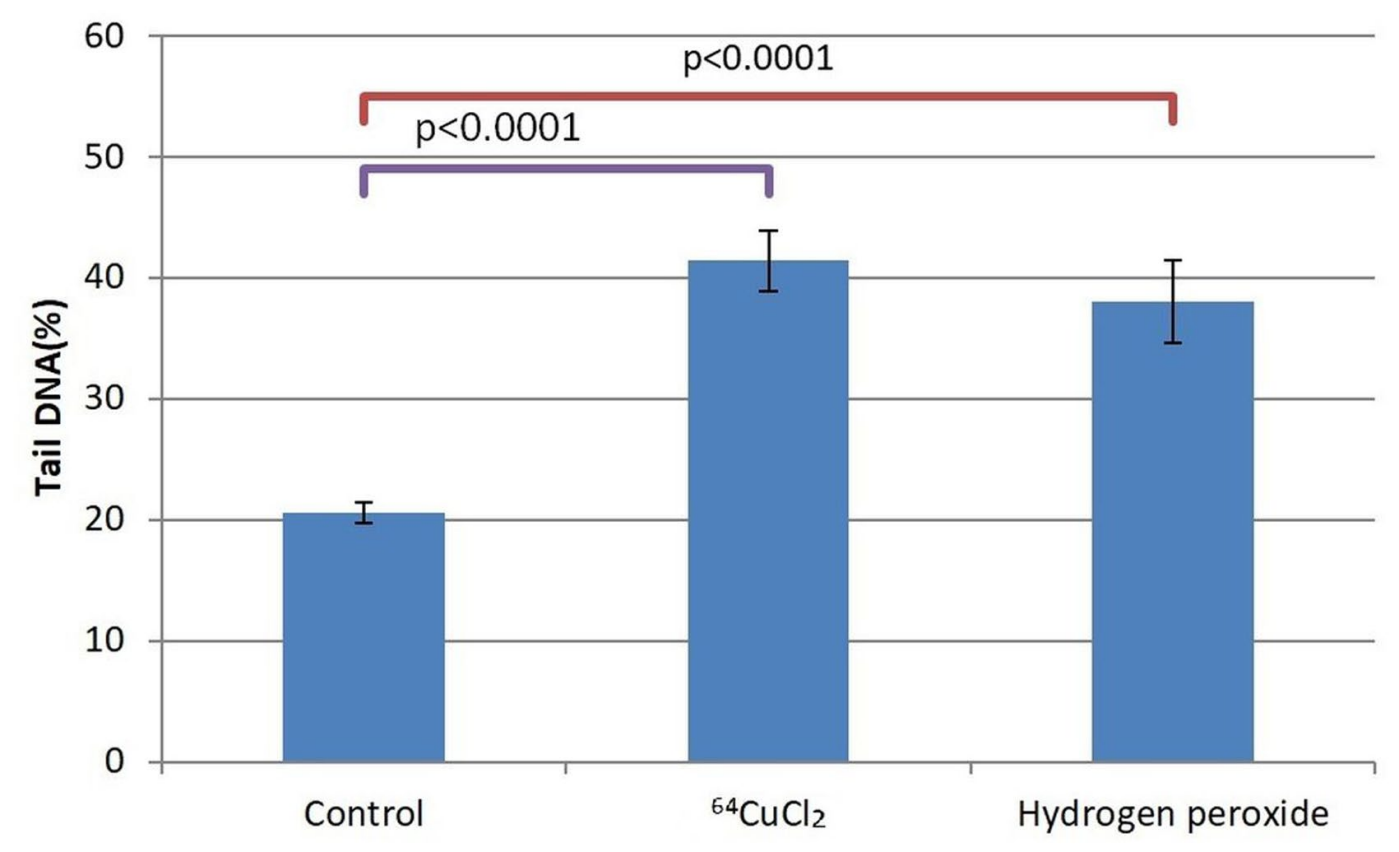
The percentage of cells in the tail DNA of HCT116 cells treated for 24 h with 20 MBq/mL ^64^Cu or 10 μM hydrogen peroxide. Evaluation was performed with the alkaline comet assay. Data are presented as mean value ± SD of the values comprised in the P_25_-P_75_ interval. Comparison between treated and untreated controls was performed with the non-parametric Mann–Whitney U test.

The genotoxic effect of 20 MBq/mL [^64^Cu]CuCl_2_ was assessed on human HCT116 colon carcinoma cells which proved to be highly reactive to the radioisotope, as demonstrated by the MTS reduction test (Figures 3, 4). DNA damage was assessed using the alkaline comet assay, through the percentage of DNA in the comet tails. Untreated cells were considered as a negative control, and cells treated with 10 μM H_2_O_2_ as a positive control (Figure 5).

Given the dispersion of the results of DNA tail percentage in each sample, we selected the P_25_-P_75_ interval for statistical analysis. As compared to untreated controls, a doubling of the DNA percentage in the comet tails corresponding to cells exposed either to 20 MBq/mL [^64^Cu]CuCl_2_ or to 10 μM H_2_O_2_ was evidenced at 24 h after initiating the treatment (*p* < 0.0001). This early genotoxic effect of ^64^CuCl_2_ on HCT116 tumor cells was associated with the decrease in the number of metabolically active HCT116 cells (Figure 4).

### 3.4. The death-inducing effects of ^64^Cu

**Figure 6:**
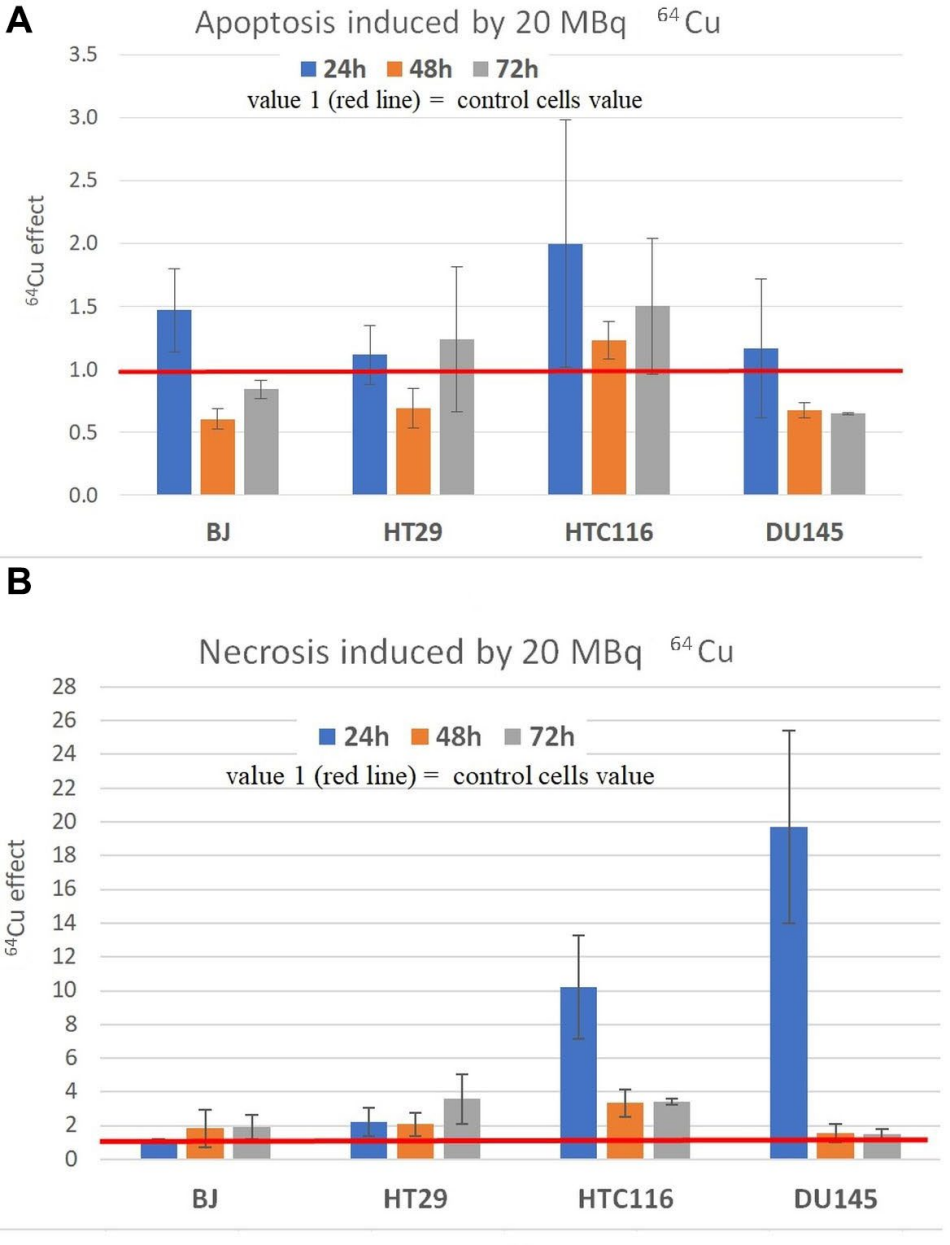
Apoptosis **(A)** and necrosis **(B)** induced by 20 MBq/mL ^64^Cu in normal human BJ fibroblasts and various human tumor cells (HT29 and HCT116 colon carcinoma, and DU145 prostate carcinoma cells). Apoptosis and necrosis were evaluated by fluorescent microscopy with acridine orange and ethidium homodimer-1. Results are expressed as mean ± SEM of the ^64^Cu effect on the percentage of apoptotic or necrotic cells for 2 independent experiments. The mean effect was calculated in each experiment as parameter value in exposed cells divided by the parameter value in unexposed control cells, and was further used for the statistics of 2 independent experiments.

The pronounced decrease of MTS reduction, and hence of the number of metabolically active cells, due to the treatment with 20 MBq/mL [^64^Cu]CuCl_2_ (Figure 4), was analyzed also from the perspective of cell death by investigating the percentage of apoptotic and necrotic cells (Figure 6). No major increase in the percentage of apoptotic cells was observed in cultures treated with [^64^Cu]CuCl_2_, with a tendency of increase only in normal BJ fibroblasts and HCT116 colon carcinoma cells at 24 h after the addition of the radioisotope (Figure 6A). Only a small increase of the percentage of necrotic cells was generally registered (1.16× and 2.22× compared to untreated cells) in the case of normal BJ fibroblast and colon adenocarcinoma HT29 cells, while for HCT116 colon carcinoma cells and DU145 prostate carcinoma cells, higher percentages (10.21× and 19.7× compared to untreated cells) of necrotic cells were detected at 24 h after the addition of the radioisotope (Figure 6B). Corroborating MTS reduction data (Figure 3) with necrosis data (Figure 6B) in the case of DU145 cells, we observed that the number of metabolically active DU145 cells was not found to decrease at 24 h as compared to non-treated cells (Figure 3A), but a higher number of necrotic cells was detected (Figure 6B). This apparent inconsistency may arise from an increase in the cell metabolism triggered by ^64^Cu in a part of DU145 cells, while other cells were driven to necrosis. Of note is that apoptosis and necrosis were measured at fixed time points (24 h, 48 h, and 72 h). Therefore, we may not exclude that a higher number of necrotic cells would have been detected earlier than 24 h after radioisotope addition, considering that necrotic cells finally disintegrate (41).

We investigated LDH release as a measure of membrane integrity in treated and untreated cells. Increased LDH release is registered when necrosis/ necroptosis occurs (42). Albeit being measured at fixed time points, the LDH release assay provides information on the whole amount of LDH released before performing the assay, unlike the apoptosis/necrosis assay data that are a snapshot of the cellular status at the moment of testing.

**Figure 7:**
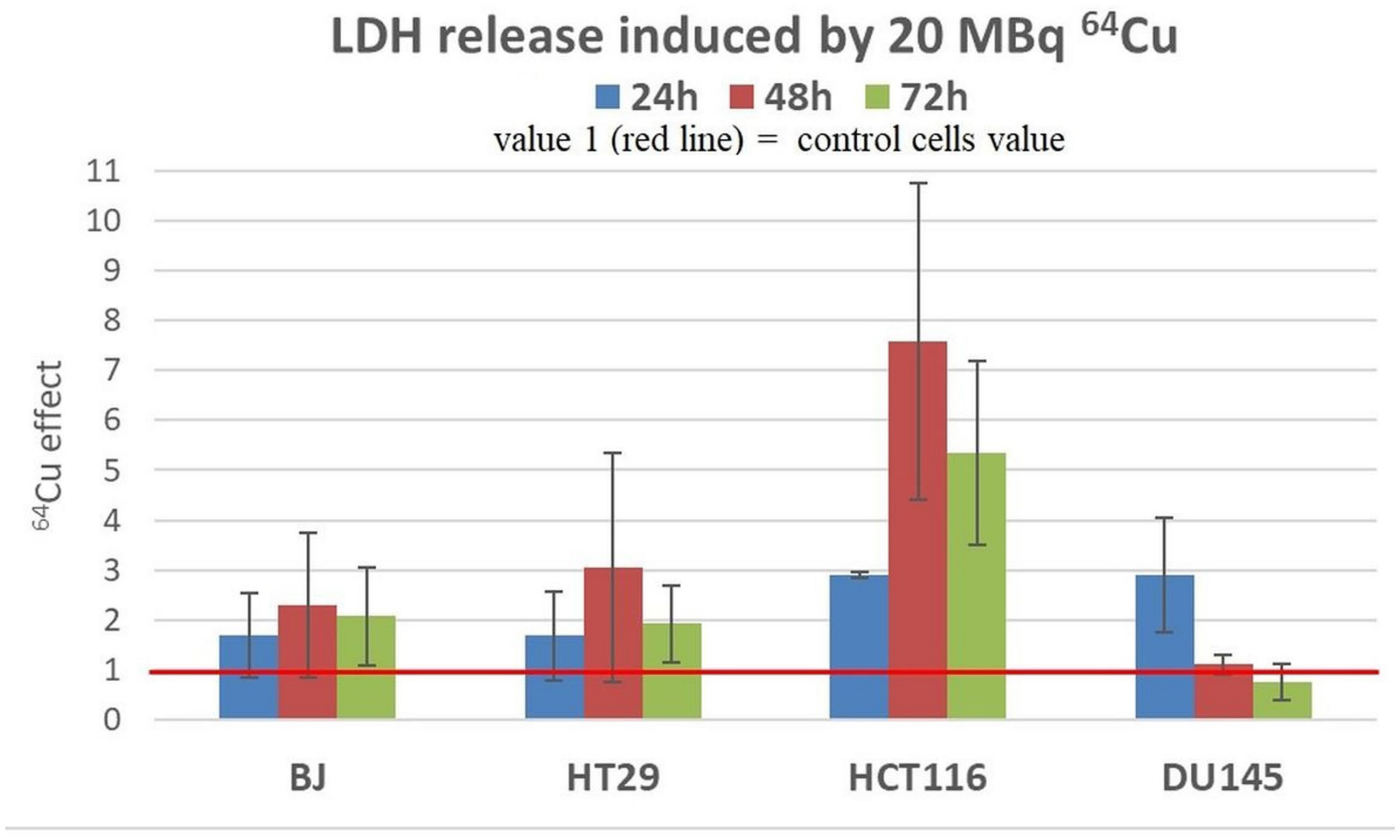
LDH release induced by 20 MBq/mL ^64^Cu in cultures of normal human BJ fibroblasts and various human tumor cells (HT29 and HCT116 colon carcinoma and DU145 prostate carcinoma cells). Results are expressed as mean ± SEM of ^64^Cu effect on the percentage of apoptotic or necrotic cells for 2 independent experiments. The mean effect was calculated in each experiment as parameter value in exposed cells divided by the parameter value in untreated controls, and was further used for the statistics of 2 independent experiments.

Results showed that a moderate increase in LDH release was registered in all the investigated cell lines exposed to 20 MBq/mL (Figure 7), indicating that ^64^Cu induces alterations of plasma membrane integrity associated with necrosis/necroptosis (43, 44). The most prominent effects of the radioisotope were detected in the case of HCT116 colon carcinoma cells at all the investigation time points, and in DU145 prostate carcinoma cells only at 24 h after radioisotope addition. The increased levels of LDH release are only partly explained by the moderate increase in the percentage of necrotic cells (Figure 7).

### 3.5. Oxidative stress markers

**Figure 8:**
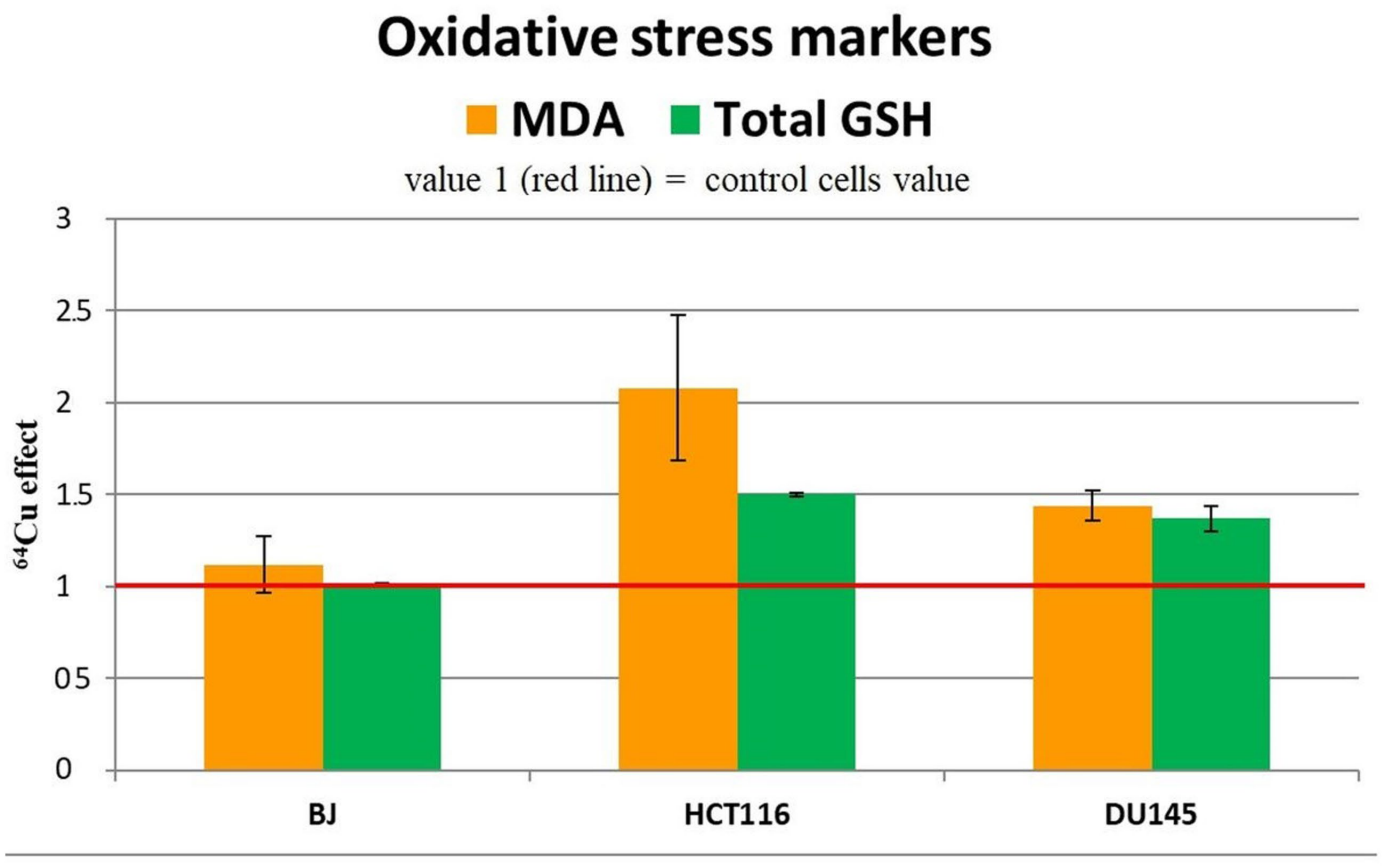
Representative data for oxidative stress markers (MDA level and total GSH) in the tumor cell lines HCT116 si DU145, and in the normal BJ cell line following 24 h incubation with 20 MBq/mL ^64^Cu. Results are expressed as mean ± SEM of ^64^Cu effect on the percentage of MDA and total GSH of treated cells compared with control for 2 independent experiments.

We analyzed the changes in the MDA and reduced glutathione (GSH) content to assess the redox status of the investigated cells from the perspective of lipid peroxidation (45) and antioxidant protection (46), respectively. An important increase of MDA and total GSH levels was induced by the treatment of HCT116 colon tumor cells with 20 MBq/mL ^64^CuCl_2_ for 24 h (Figure 8), indicating that ^64^Cu triggered in these cells not only DNA damage (Figure 5), but also oxidative stress, both contributing to the decrease in the number of metabolically active HCT116 cells (Figure 4) following ^64^Cu exposure. Results also show that the increased GSH levels could not completely protect HCT116 cells against the radioisotope-induced oxidative damage.

Increased MDA and GSH levels were registered also in DU145 prostate carcinoma cells treated for 24 h with 20 MBq/mL [^64^Cu]CuCl_2_ (Figure 8), which were associated with an increase in the number of metabolically active cells (Figure 4). The oxidative stress was more prominent in HCT116 cells which were more affected by the ^64^Cu treatment than DU145 tumor cells, in terms of the decrease of the number of metabolically active cells (Figure 4), while GSH level changes were almost similar (Figure 8).

The increase in the antioxidant response indicated that oxidative stress occurs, as also shown by the elevated MDA levels, which cells attempt to counteract through the increase of GSH, the major antioxidant buffer of cells (47).

Meanwhile, in normal BJ fibroblasts, ^64^Cu did not induce redox changes (Figure 8), indicating a different action of the radioisotope in normal and tumor cells. In the case of normal BJ cells, the basal level of glutathione seems to be sufficient to counteract the potential oxidative stress induced by ^64^Cu.

### 3.6. Gene expression

For characterizing cellular responses to ^64^CuCl_2_, the expression level of 84 stress genes (Supplementary File 1) was investigated by qRT-PCR in tumor and normal cells exposed for 24 h to the [^64^Cu]CuCl_2_ solution (20 MBq/mL).

The biologic pathways in which these genes are involved is described in Supplementary File 1 (Table 1), according to Qiagen Geneglobe.^1^ More details on the investigated stress genes can be found online in GeneCards: Human Gene Databse.^2^

**Figure 9:**
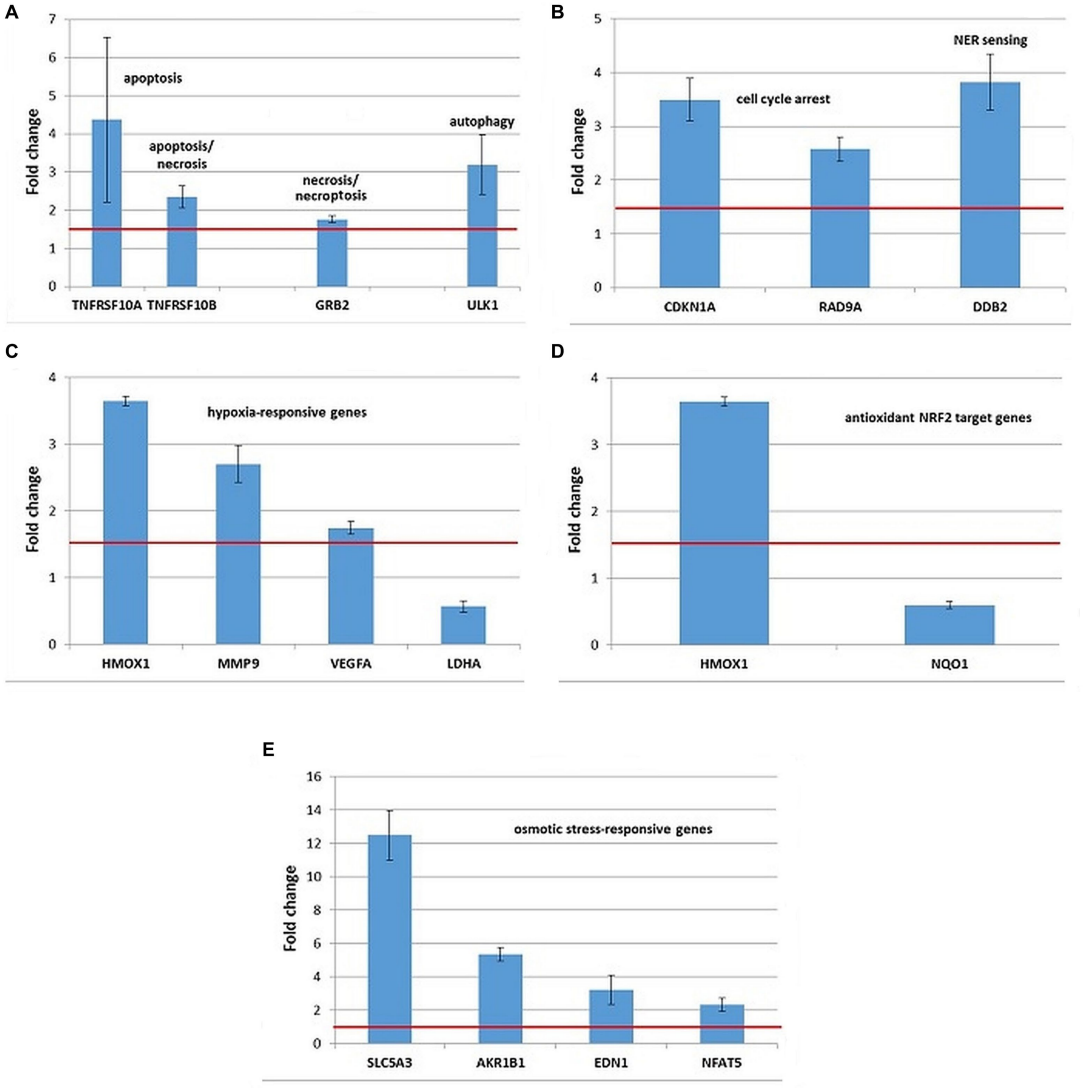
Changes in the expression of stress genes triggered by the treatment of human HCT116 colon carcinoma cells with 20 MBq/mL ^64^Cu for 24 h. **(A)** Cell death; **(B)** DNA damage and repair; **(C)** hypoxia response; **(D)** antioxidant response; **(E)** osmotic stress. qRT-PCR results were presented as mean value of Fold Change (FC) ± SEM for 3 independent experiments. Only genes with significant expression changes (1.5 < FC < 0.6) were represented.

**Figure 10:**
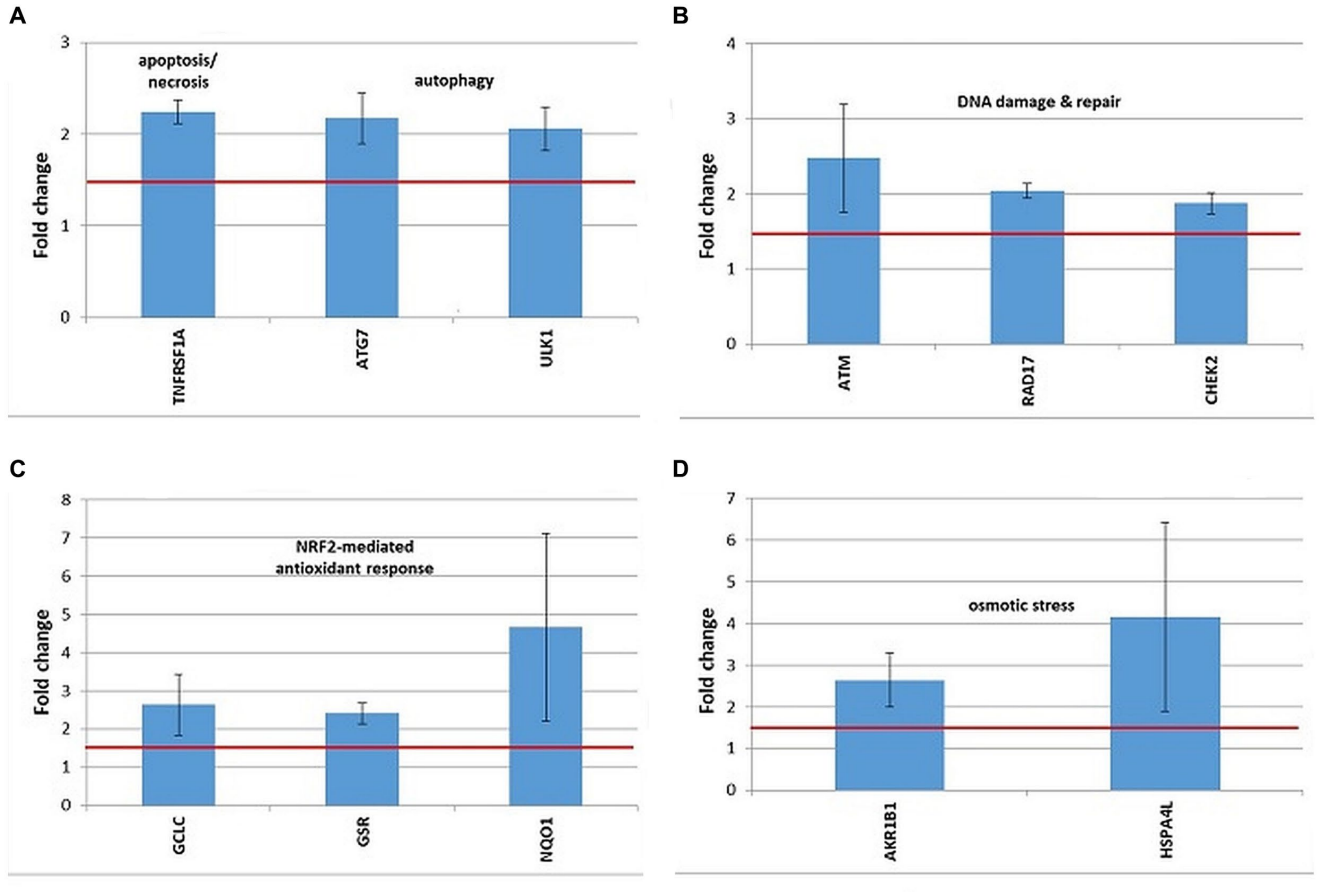
Changes in the expression of stress genes triggered by the treatment of human DU145 prostate carcinoma cells with 20 MBq/mL ^64^Cu for 24 h. **(A)** Cell death; **(B)** DNA damage and repair; **(C)** antioxidant response; **(D)** osmotic stress. qRT-PCR results were presented as mean value of Fold Change (FC) ± SEM for 3 independent experiments. Only genes with significant expression changes (1.5 < FC < 0.6) were represented.

**Figure 11:**
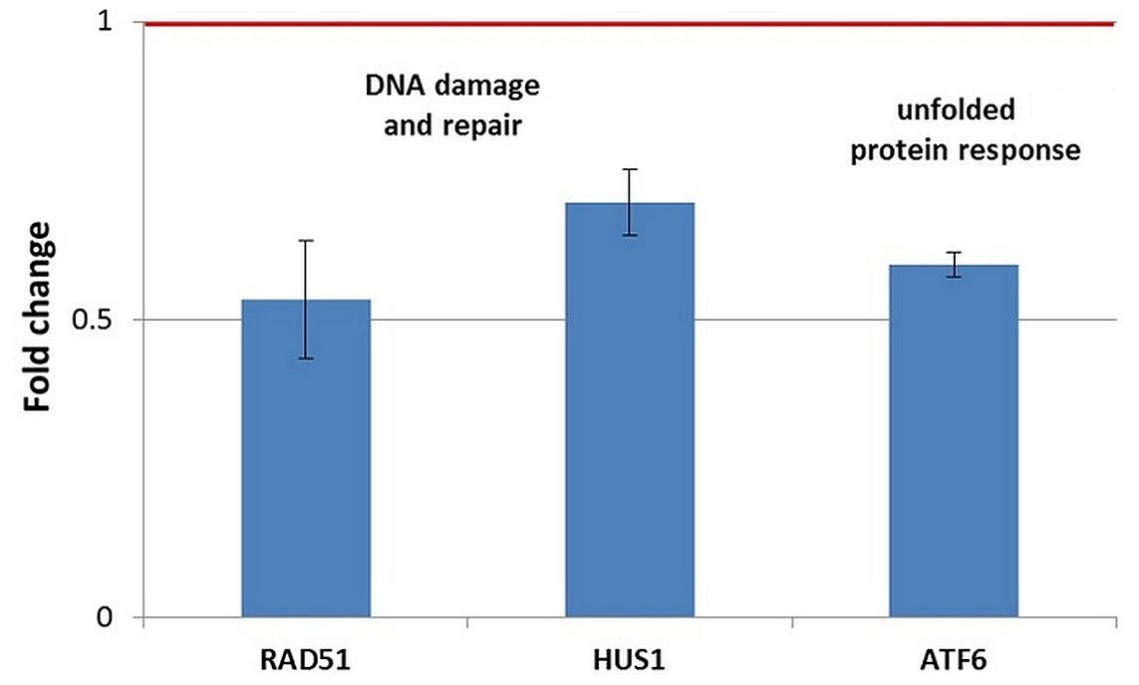
Changes in the expression of stress genes triggered by the treatment of human normal BJ fibroblasts with 20 MBq/mL ^64^Cu for 24 h. qRT-PCR results were presented as mean value of Fold Change (FC) ± SEM for 3 independent experiments. Only genes with significant expression changes (1.5 < FC < 0.7) were represented.

Stress genes that were consistently upregulated (FC > 1.5) in 3 independent experiments were represented in Figures 9–11, but comments on genes with expression changes in 2 out of 3 experiments were made in the text.

HCT116 tumor cells exposed to 20 MBq/mL [^64^Cu]CuCl_2_ for 24 h showed overexpression of some genes involved in cell death by apoptosis (TNFRSF10A, TNFRSF10B, and BBC3), necrosis/necroptosis (GRB2 and TNFRSF10A) and autophagy (ULK1), as shown in Figure 9A. The TNFRSF1 gene involved both in apoptosis and necrosis was found upregulated only in 2 out of the 3 independent experiments (FC = 2.87 ± 1.21).

The upregulation of the CDKN1A and DDB2 genes (Figure 9B) involved in DNA damage and repair indicated that the death of HCT116 cells was partially underlined by a genotoxic action of ^64^Cu, as also shown by the results of the Comet assay (Figure 5). The TP53 gene, critical for the DNA damage responses was found upregulated only in 2 out of the 3 independent experiments (FC = 2.3 ± 0.49).

No consistent results were obtained regarding the potential proteotoxic effects of the treatment with [^64^Cu]CuCl_2_, as the DNAJC3 and HSPA4 genes were found over-expressed only in 2 out of the 3 independent experiments (data not shown), and no significant expression changes were registered in other investigated unfolded protein response-related genes.

Exposure of HCT116 tumor cells for 24 h to ^64^CuCl_2_ induced the upregulation of some hypoxia-responsive genes such as the HMOX1, MMP9, and VEGFA, the most striking overexpression being registered in the case of the HMOX1 gene (Figure 9C). Meanwhile, the hypoxia-responsive LDHA gene was found down-regulated (Figure 10C) at 24 h after the initiation of the *in vitro* treatment.

Considering that HMOX1 is involved not only in hypoxia but also in the antioxidant response mediated by the transcription factor NRF2 (48), the expression of several NRF2 target genes was investigated (Supplementary File 1). Even though both HMOX1 and NQO1 are validated NRF2 targets (49), results evidenced the downregulation of the NQO1 gene in HCT116 cells treated with [^64^Cu]CuCl_2_, while HMOX1 was found clearly upregulated (Figure 9D). Moreover, we found that the NRF2 target genes GCLM and GCLC involved in glutathione biosynthesis (50) were upregulated only in 2 of the 3 independent experiments. Accordingly, the stress genes expression pattern was inconclusive regarding the involvement of the NRF2 transcription factor in the response of HCT116 tumor cells to [^64^Cu]CuCl_2_ treatment, at least at 24 h after radioisotope addition in cell cultures.

Several genes involved in cellular responses to osmotic stress were evidenced in HCT116 cells exposed to ^64^CuCl_2_, such as AKR1B1, NFAT5, SLC5A3, and EDN1 (Figure 9E), the most striking upregulation being registered in the case of the SLC5A3 gene.

A distinctive expression pattern of stress genes was obtained in human prostate carcinoma DU145 cells treated for 24 h with 20 MBq ^64^Cu (Figure 10), which only partially overlapped the pattern evidenced in human colon carcinoma HCT116 cells (Figure 9).

In DU145 cells, the over-expressed cell death-related genes were TNFRSF1, involved in apoptosis and necrosis, along with ATG7 and ULK1, involved in autophagy (Figure 10A). Only ULK1 was found to be upregulated both in DU145 and HCT116 cells (Figure 9A).

DNA damage was evidenced by the upregulation of the ATM gene, involved in DNA damage sensing (51), along with the RAD17 and CHEK1 genes, involved in cell cycle arrest (Figure 10B). In the case of DU145, no conclusive results were obtained regarding the overexpression of the CDKN1A gene, participating in cell cycle arrest, which was found upregulated only in 2 out of the 3 independent experiments (FC = 3.52 ± 1.45). Meanwhile, this gene was clearly over-expressed in HCT116 cells in 3 independent experiments (Figure 9B).

The oxidative stress elicited by the treatment of DU145 cells with [^64^Cu]CuCl_2_ was highlighted by the upregulation of the antioxidant GCLC and GSR genes involved in glutathione biosynthesis (52) and metabolism (53), respectively (Figure 10C). The antioxidant NQO1 gene (54) was as well over-expressed in DU145 cells (Figure 10C). Considering that GCLC, GSR, and NQO1 are targets of the cytoprotective NRF2 transcription factor, results indicate that the NRF2 signaling pathway was activated by [^64^Cu]CuCl_2_ treatment in DU145 cells, probably in response to an elicited oxidative stress. Meanwhile, in the case of HCT116 cells the NRF2 fingerprint was elusive, as only the NRF2 target gene HMOX1 (55) was found up-regulated, while NQO1 was down-regulated following the treatment of cells with ^64^CuCl_2_ (Figure 9D).

The exposure of DU145 cells to ^64^Cu did not induce a consistent overexpression of stress genes involved in hypoxia responses (data not shown), unlike HCT116 tumor cells for which ^64^CuCl_2_ induced the upregulation of the hypoxia-responsive genes HMOX1 and MMP9, as presented in Figure 9C.

Some genes related to osmotic stress were over-expressed in DU145 cells (Figure 10D), such as AKR1B1, which was also found upregulated in HCT116 cells (Figure 10E), and HSPA4L, which was over-expressed also in HCT116 cells, but only in 2 out of the 3 independent experiments (FC = 2.51 ± 0.11). Very dispersed expression changes were registered in independent experiments on DU145 cells in the case of the SLC5A3 gene (FC = 14.68 ± 9.95), while consistent upregulation was registered in HCT116 cells following exposure to ^64^Cu (Figure 9E).

In normal BJ fibroblasts exposed for 24 h to ^64^CuCl_2_ (Figure 11), only a few stress genes with important expression changes were identified. The genes with consistent downregulation in 3 independent experiments were the DNA damage-related genes RAD51 and, to a lesser extent, HUS1, as well as the ATF6 gene involved in the unfolded protein response (Figure 11). The only upregulated stress gene was SLC5A3 related to osmotic stress (FC = 14.89 ± 9.16). This pattern of gene expression changes was observed exclusively in normal BJ fibroblasts.

A comparison between the expression pattern of stress genes at 24 h after the addition of [^64^Cu]CuCl_2_ was performed in the two investigated colon carcinoma cell lines HCT116 and HT29 (Supplementary File 1; Table 2). A lower number of stress genes had significant expression changes in HT29 cells, as compared to HCT116 cells, mainly regarding cell death, genotoxic, proteotoxic, oxidative, and osmotic stress. Meanwhile, in both HCT116 and HT29 colon tumor cells treated with [^64^Cu]CuCl_2_ an important fingerprint of hypoxia was registered through the upregulation of HMOX1, MMP9, and SERPINE1. Downregulation of CA9 and upregulation of SCL2A1 were exclusively observed in HT29 cells. Several genes related to other signaling pathways were over-expressed in both of the colon tumor cells under investigation: RIPK1, related to necroptosis (56), CDKN1A with a critical role in cell cycle arrest, GCLM, involved in glutathione biosynthesis, and SCL5A3 related to cellular responses to osmotic stress. Moreover, NQO1 was down-regulated both in HCT116 and HT29 cells, unlike the case of DU145 cells for which the upregulation of NQO1 was registered (Figure 10C). Results indicate a strong stress response at 24 h after the addition of [^64^Cu]CuCl_2_ in HCT116 cells that are more reactive to the treatment than HT29 cells in terms of MTS reduction (Figure 4).

**Table 2:** Stress genes with significant expression changes in HCT 116 and HT29 colon carcinoma cells treated for 24 h with 20 MBq/mL ^64^CuCl_2_.

Pathway / Gene	Colon carcinoma cells	
	HCT 116	HT29
	*FC value*	*FC value*
*Cell death*		
FAS	4.99 ± 1.99	
TNFRSF1A	1.67 ± 0.28	
TNFRSF10B	2.61 ± 0.18	
TNFRSF10A	2.23 ± 0.56	
BBC3	3.53 ± 0.13	
RIPK1	2.07 ± 0.18	2.07 ± 0.33
*Genotoxic stress*		
ATM	1.79 ± 0.05	
RAD17	1.71 ± 0.03	
RAD9A	2.39 ± 0.23	
HUS1	2.11 ± 0.27	
DDB2	3.70 ± 087	
CDKN1A	3.35 ± 0.63	3.18 ± 1.04
*Proteotoxic stress (unfolded protein response)*		
BBC3	3.53 ± 0.13	
DNAJC3	2.42 ± 0.55	
HSPA4	1.79 ± 0.10	
CALR		1.54 ± 0.03
*Hypoxic stress*		
HMOX1	3.71 ± 0.00	2.85 ± 0.75
MMP9	2.62 ± 0.46	4.50 ± 0.26
SERPINE1	2.57 ± 1.04	2.28 ± 0.44
VEGFA	1.78 ± 0.15	
SLC2A1		1.97 ± 0.25
CA9		0.54 ± 0.10
*Oxidative stress (NRF2-mediated antioxidant response)*		
HMOX1	3.71 ± 0.00	2.85 ± 0.75
GCLC	2.15 ± 0.25	
GCLM	2.06 ± 0.44	2.09 ± 0.25
PRDX1	1.78 ± 0.07	
NQO1	0.63 ± 0.07	
*Osmotic stress*		
AKR1B1	5.10 ± 0.53	
SLC5A3	13.90 ± 0.76	19.83 ± 8.67
EDN1	2.99 ± 1.43	
NFAT5	2.55 ± 0.56	
HSPA4L	2.51 ± 0.11	

Data from 2 independent experiments were expressed as FC values ± SEM. Only genes with FC values above 1.5 or below 0.6 were presented.

Altogether, it appears that fewer stress genes exhibited modified expression at 24 h after ^64^CuCl_2_ addition in those cell lines that were less reactive to radioisotope exposure in terms of MTS reduction, such as normal BJ fibroblasts and HT29 colon adenocarcinoma (Supplementary File S1; Table 3). The lack of expression changes for critical stress genes or their downregulation at 24 h indicate that transcription might have occurred earlier, during the first 24 h after [^64^Cu]CuCl_2_ addition. It is possible that less reactive cells attempted to terminate the stress response at 24 h, probably after repairing some of the cellular damages inflicted by the radioisotope (57).

**Table 3:** Stress genes with modified expression in human tumor cells (colon carcinoma HCT116 cells, colon adenocarcinoma HT29 cells and prostate carcinoma DU145 cells) and human normal BJ fibroblasts.

Pathway	Genes	Cells			
		*HCT116*	HT29*	*DU145*	*BJ*
Cell death	ATG7	−	−	+	−
	GRB2	+	−	−	−
	RIPK1	+/−	+	+/−	−
	TNFRSF10A	+	−	−	−
	TNFRSF10B	+	−	−	−
	TNFRSF1A	+/−	+	+	−
	ULK1	+	−	+	−
DNA damage& repair	ATM	−	−	+	−
	CDKN1A	+	+	+/−	+/−
	CHEK2	−	−	+	−
	DDB2	+	−	−	−
	HUS1	+/−	−	−	+**
	RAD17	+/−	−	+	−
	RAD51	−	−	−	+**
	RAD9A	+	−	−	−
Unfolded protein response	ATF6	−	−	−	+**
	BBC3	+/−	−	−	−
	CALR	−	+	−	−
	DNAJC3	+/−	−	−	−
	HSPA4	+/−	−	−	−
Hypoxic stress	CA9	−	+	−	−
	HMOX1	+	+	+/−	−
	LDHA	+**	−	+/−	−
	MMP9	+	+	−	−
	SERPINE1	+	+	−	−
	SLC2A1	−	+	+/−	−
	VEGFA	+	−	−	−
Antioxidant response	GCLC	+/−		+	
	GCLM	+/−	+	+/−	−
	GSR	−	−	+	−
	HMOX1	+	+	−	−
	NQO1	+**	−	+	−
	PRDX1	+/−	−	+/−	−
Osmotic stress	AKR1B1	+	+/−	+	−
	EDN1	+	−	−	−
	HSPA4L	+	−	+	−
	NFAT5	+	−	−	−
	SLC5A3	+	+	+	+

With + was marked the case when a gene had similar expression changes in 3 independent experiments; with +/− was marked the case when a gene had similar expression changes in 2 out of the 3 independent experiments; *HT29 cells were investigated only in 2 independent experiments; **Down-regulated genes.

## 4. Discussions

The study aimed to analyze both the damages inflicted by ^64^CuCl_2_ and by the radioactive decay emissions (β^−^ and Auger electrons) of ^64^Cu, in human colon (HT29 and HCT116) and prostate carcinoma (DU145) cell lines, as well as in human normal BJ fibroblasts and the associated stress responses underlying cytotoxic and repair mechanisms in exposed cells. Starting from the level of activities usually used in PET imaging scanning (39, 40), we tested gradually increased radioactive concentrations of ^64^Cu, from 2 MBq/mL up to 40 MBq/mL, for investigating the potential therapeutic effects of this radionuclide.

All the investigated cells incorporated ^64^Cu similarly within a couple of hours, probably through Cu receptors (58), independent of their tumoral or normal status. Instead, their fate after exposure to ^64^CuCl_2_ was cell-dependent due to intrinsic differences in cellular responses against the stress inflicted by the radionuclide (59).

A decrease in the number of metabolically active cells was registered by the MTS reduction test at 24 h after [^64^Cu]CuCl_2_ addition and was persistent for 72 h. The investigated normal and tumor cell lines had distinctive reactivity to ^64^CuCl_2_ in terms of the inhibitory effects exerted by 20 MBq/mL [^64^Cu]CuCl_2_ on MTS reduction: HCT116 human colon carcinoma > DU145 human prostate carcinoma > HT29 human colon adenocarcinoma > normal human BJ fibroblasts. Albeit being less affected by lower radioactive concentration of ^64^Cu (2 and 10 MBq/mL) as compared to tumor cells, normal BJ fibroblasts were disturbed by higher activities of the radioisotope, indicating an unwanted radiotoxic effect of ^64^CuCl_2_.

The persistent effects of ^64^CuCl_2_ on MTS reduction suggest that a dynamic equilibrium between cell death and proliferation might take place. We cannot rule out the contribution of cell cycle arrest in the persistent decrease of MTS reduction, as an important overexpression of some pathway-related genes was registered at 24 h after radioisotope addition, such as CDKN1A in HCT116 and HT29 cells, RAD9A in HCT116 cells, along with CHEK2 and RAD17A in DU145 cells.

The most striking inhibitory effects on the MTS reduction were registered in the human colon carcinoma HCT116 cell line treated with ^64^CuCl_2_. In this case, the number of metabolically active cells decreased almost to zero in 24 h after exposure to the high ^64^Cu radioactive concentration of 40 MBq/mL, indicating a potential therapeutic effect of ^64^CuCl_2_. A lower activity, corresponding to 20 MBq/mL radioactive concentration, decreased MTS reduction to ~30% compared to control (untreated cells), and was further used for mechanistic studies. The inhibitory effect was underlined by DNA damage, and was accompanied by the upregulation of critical genes involved in DNA repair through cell cycle arrest (CDKN1A and RAD9A) (60, 61) or nucleotide excision repair (DDB2) (62). According to gene expression data, the death of treated HCT116 cells occurred through extrinsic apoptosis (63) mediated by the death receptors DR4 and DR5 encoded by the TNFRSF10A and TNFRSF10B genes, respectively. The overexpression of the GRB2 gene indicated that part of the HCT116 cells was engaged in necrosis/necroptosis (64), while the overexpression of ULK1 evidenced the occurrence of autophagy (65) at 24 h after radioisotope addition.

Oxidative stress was inflicted by 20 MBq/mL [^64^Cu]CuCl_2_ in HCT116 colon carcinoma and DU145 prostate carcinoma cells, but no fingerprint of oxidative stress was registered in the less reactive normal BJ fibroblasts. The upregulation of the ULK1 gene evidenced a mechanism of ROS-induced cell death in HCT116 and DU145 cells (66), but antioxidant genes were as well overexpressed, suggesting that these tumor cells attempted to counteract the oxidative damages induced by ^64^Cu. In the case of HCT116 tumor cells, the antioxidant response was mediated by increased GSH levels and by the overexpression of the antioxidant gene HMOX1 involved also in iron metabolism and hypoxia responses, which is at the crossroad of several transcription factors, including the cytoprotective NRF2 transcription factor (67). Meanwhile, NQO1, which is also a validated NRF2 target (68), was found downregulated in HCT116 cells, questioning the activation status of NRF2 at 24 h after radioisotope addition. The weaker antioxidant response mediated by NRF2 may partially account for the marked decrease in the number of metabolically active HCT116 cells due to their exposure to ^64^Cu. Nevertheless, a similar NQO1 downregulation observed in HT29 colon adenocarcinoma cells was not associated with a comparable decrease of MTS reduction, suggesting that other mechanisms than those mediated by NRF2 might protect HT29 cells against the damages inflicted by ^64^Cu. Meanwhile, in the less reactive DU145 prostate carcinoma cell line, NQO1 was found upregulated. These cells presented lower levels of lipid peroxidation in comparison with HCT116 cells, and similar levels of GSH, as well as an increased expression of the NQO1, GCLC, and GCLM genes targeted by NRF2. This pattern of the antioxidant stress response, partly mediated by NRF2, may account for the increased survival of treated DU145 cells in comparison with HCT116 cells in the context of lower lipid peroxidation levels.

An important fingerprint of hypoxia (69) was registered in HCT116 cells at 24 h after the addition of 20 MBq/mL [^64^Cu]CuCl_2_, characterized by the overexpression of the HMOX1 (70) and MMP9 (71) genes that can elicit hypoxic radioresistance (72) in the tumor niche (73). Meanwhile, no consistent molecular fingerprint of hypoxia was detected in the less reactive DU145 cells, indicating that either the levels of radiation-induced hypoxia were lower than in the case of HCT116 cells, or DU145 cells managed to cope with the damages inflicted by ^64^Cu within the first 24 h after radioisotope addition.

Surprisingly, exposure of all the investigated cell types to ^64^Cu induced the massive overexpression of the SLC5A3 gene which encodes the Myo-inositol co-transporter that regulates cellular responses to osmotic stress (74). Apparently, ^64^CuCl_2_ solution in PBS might be responsible for hypertonic stress (75), although the amount added to cell cultures was too low to sustain this hypothesis. The mechanism of radiation-induced osmotic stress should be further investigated.

The main limitation of the *in vitro* study is related to the fact that cellular models may not be able to reproduce the results observed *in vivo* on animal models or in cancer patients. As Auger-Meitner electrons are very-short range particles with a very high linear energy transfer, the *in vitro* model completely immersed in an aqueous medium limits the translation of *in vitro* results to complex organisms.

Concluding, the *in vitro* study brings new insights into the molecular mechanisms underlying the therapeutic action of ^64^Cu in solid tumors. ^64^CuCl_2_ with a radioactive concentration of 40 MBq/mL appears to have therapeutic effects in colon carcinoma, but its use is limited by harmful, yet lower effects on normal fibroblasts. A lower radioactive concentration of 10–20 MBq/mL might have a partial therapeutic effect on tumor cells, but with less radiotoxicity in normal fibroblasts. Colon carcinoma cells attempt to counteract the damaging effects of radiation but could only partially cope with the radioactive cue. Meanwhile, normal human BJ fibroblasts were less reactive or managed to repair damages within the first 24 h after the addition of ^64^CuCl_2_.

Understanding the molecular mechanisms by which the ^64^Cu differentially affect tumor and normal cells, along with the elicited stress responses, can help to design an improved theragnostic strategy in cancer. The study provides first-time evidence on the complex stress responses triggered by ^64^CuCl_2_ in various human tumor and normal cells, bringing into focus the hypothesis that the therapeutic efficacy of ^64^CuCl_2_ might be increased by specifically inhibiting particular repair mechanisms.

## Data availability statement

The original contributions presented in the study are included in the article/supplementary materials, further inquiries can be directed to the corresponding author/s. Gene expression data were deposited at: https://doi.org/10.7910/DVN/TDMUTY, Harvard Dataverse, V1.

## Author contributions

DN designed and participated in the radiochemistry studies, assured logistics and funding, and reviewed the manuscript. GM organized and coordinated the biological studies, edited the results and discussions, and reviewed the manuscript. RMS coordinated and participated in all the experiments and study methodologies, wrote the methods, and results chapters. AD reviewed the results and discussions. RAL and DC prepared the targets and radioactive solutions. LEC and MRC performed the quality control of radioactive solutions and participated to binding assay. MT and CM prepared the cell cultures and performed the genotoxicity assays. IN and DAN conducted the viability tests. IN, DAN, and MD performed the genotoxicity and gene expression assays. MS and AD performed the oxidative-stress assays. All authors contributed to the article and approved the submitted version.

## Funding

This work was supported by a grant of the Ministry of Research, Innovation and Digitization, CNCS/CCCDI – UEFISCDI project number ERANET-EURONAMOMED-3-I2PAD, within PNCDI III and partially sustained through the Nucleu grants PN 23.16.02.01/2023 and PN 23.21.02.01/2023.

## Conflict of interest

The authors declare that the research was conducted in the absence of any commercial or financial relationships that could be construed as a potential conflict of interest.

## Publisher’s note

All claims expressed in this article are solely those of the authors and do not necessarily represent those of their affiliated organizations, or those of the publisher, the editors and the reviewers. Any product that may be evaluated in this article, or claim that may be made by its manufacturer, is not guaranteed or endorsed by the publisher.

## Footnotes

^1^https://geneglobe.qiagen.com/us/product-groups/rt2-profiler-pcr-arrays

^2^https://www.genecards.org/
