# Supplementary material for: Modifications in cellular viability, DNA damage and stress responses inflicted in cancer cells by copper-64 ions
Source: Front Med (Lausanne). 2023 Jun 21;10:1197846. doi: 10.3389/fmed.2023.1197846 (PMC10320858; doi:10.3389/fmed.2023.1197846)
Supplement: Supplementary file 1 [file Table_1.DOCX]

Supplementary Material

Article Title

**Radu M. Serban, Dana Niculae*, Gina Manda*, Ionela Neagoe, Maria Dobre, Dragos A. Niculae, Mihaela Temelie, Cosmin Mustaciosu, Radu A. Leonte, Livia E. Chilug, Maria R. Cornoiu, Diana Cocioaba, Miruna Stan and Anca Dinischiotu**

**Correspondence:** Corresponding Authors: [dana.niculae@nipne.ro](mailto:dana.niculae@nipne.ro), [gina.manda@gmail.com](mailto:gina.manda@gmail.com)

# Supplementary Data


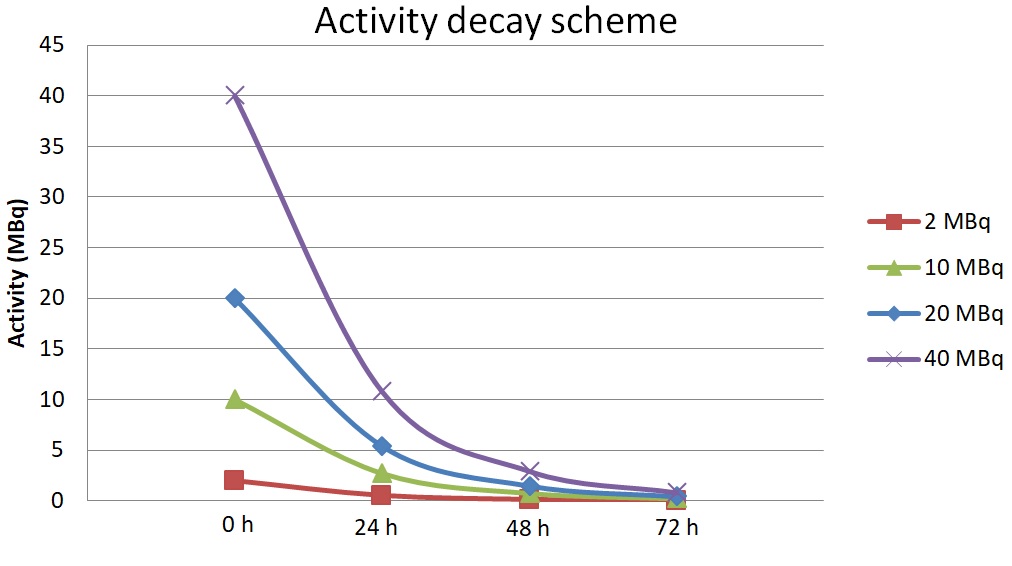


**Figure 1.** The radioactive decay of ^64^Cu activity incubated with the cell lines.

**Table 1.** The 84 stress genes analyzed

| **DNA DAMAGE & REPAIR** | | | | |
| --- | --- | --- | --- | --- |
| ***Cell Cycle Arrest & Checkpoints***: CDKN1A (p21CIP1, WAF1), CHEK1, CHEK2 (RAD53), DDIT3 (GADD153, CHOP), HUS1, MRE11, NBN, RAD17, RAD9A | | ***Other DNA Damage Responses***: ATM, ATR, DDB2, GADD45A, GADD45G, RAD51, TP53 (p53), XPC. | | |
| ***Unfolded Protein Response***: ATF4, ATF6, ATF6B, BBC3 (PUMA), BID, CALR, DDIT3 (GADD153, CHOP), DNAJC3, HSP90AA1, HSP90B1, HSPA4 (HSP70), HSPA5 (GRP78). | | | | |
| **CELL DEATH** | | | | |
| ***Apoptosis***: CASP1 (ICE), FAS, MCL1, TNFRSF10A (TRAIL-R), TNFRSF10B (DR5), TNFRSF1A (TNFR1) | ***Necrosis***: FAS, GRB2, PARP1 (ADPRT1), PVR, RIPK1, TNFRSF10A (TRAIL-R) | | | ***Autophagy***: ATG12, ATG5, ATG7, BECN1, FAS, ULK1 |
| **OXIDATIVE STRESS**: FTH1, GCLC, GCLM, GSR, GSTP1, HMOX1, NQO1, PRDX1, SQSTM1, TXN, TXNRD1 | | | **HIPOXIA SIGNALING**: ADM, ARNT, BNIP3L, CA9, EPO, HMOX1, LDHA, MMP9, SERPINE1 (PAI-1), SLC2A1, VEGFA | |
| **OSMOTIC STRESS**: AKR1B1, AQP1, AQP2, AQP4, CFTR, EDN1, HSPA4L (OSP94), NFAT5, SLC5A3 | | | **INFLAMMATORY RESPONSE**: CCL2 (MCP-1), CD40LG, CRP, CXCL8 (IL8), IFNG, IL1A, IL1B, IL6, TLR4, TNF | |

**Table 2**. Stress genes with significant expression changes in HCT 116 and HT29 colon carcinoma cells treated for 24 h with 20 MBq/mL ^64^CuCl_2_. Data from 2 independent experiments were expressed as FC values ± SEM. Only genes with FC values above 1.5 or below 0.6 were presented.

| **Pathway / Gene** | **Colon carcinoma cells** | |
| --- | --- | --- |
|  | **HCT 116** | **HT29** |
|  | ***FC value*** | ***FC value*** |
| ***Cell death*** | | |
| FAS | 4.99 ± 1.99 |  |
| TNFRSF1A | 1.67 ± 0.28 |  |
| TNFRSF10B | 2.61 ± 0.18 |  |
| TNFRSF10A | 2.23 ± 0.56 |  |
| BBC3 | 3.53 ± 0.13 |  |
| RIPK1 | 2.07 ± 0.18 | 2.07 ± 0.33 |
| ***Genotoxic stress*** | | |
| ATM | 1.79 ± 0.05 |  |
| RAD17 | 1.71 ± 0.03 |  |
| RAD9A | 2.39 ± 0.23 |  |
| HUS1 | 2.11 ± 0.27 |  |
| DDB2 | 3.70 ± 087 |  |
| CDKN1A | 3.35 ± 0.63 | 3.18 ± 1.04 |
| ***Proteotoxic stress (unfolded protein response)*** | | |
| BBC3 | 3.53 ± 0.13 |  |
| DNAJC3 | 2.42 ± 0.55 |  |
| HSPA4 | 1.79 ±0.10 |  |
| CALR |  | 1.54 ± 0.03 |
| ***Hypoxic stress*** | | |
| HMOX1 | 3.71 ± 0.00 | 2.85 ± 0.75 |
| MMP9 | 2.62 ± 0.46 | 4.50 ± 0.26 |
| SERPINE1 | 2.57 ± 1.04 | 2.28 ± 0.44 |
| VEGFA | 1.78 ± 0.15 |  |
| SLC2A1 |  | 1.97 ± 0.25 |
| CA9 |  | 0.54 ± 0.10 |
| ***Oxidative stress (NRF2-mediated antioxidant response)*** | | |
| HMOX1 | 3.71 ± 0.00 | 2.85 ± 0.75 |
| GCLC | 2.15 ± 0.25 |  |
| GCLM | 2.06 ± 0.44 | 2.09 ± 0.25 |
| PRDX1 | 1.78 ± 0.07 |  |
| NQO1 | 0.63 ± 0.07 |  |
| ***Osmotic stress*** | | |
| AKR1B1 | 5.10±0.53 |  |
| SLC5A3 | 13.90 ± 0.76 | 19.83 ± 8.67 |
| EDN1 | 2.99 ± 1.43 |  |
| NFAT5 | 2.55 ± 0.56 |  |
| HSPA4L | 2.51 ± 0.11 |  |

**Table 3.** Stress genes with modified expression in human tumor cells (colon carcinoma HCT116 cells, colon adenocarcinoma HT29 cells and prostate carcinoma DU145 cells) and human normal BJ fibroblasts. With + was marked the case when a gene had similar expression changes in 3 independent experiments; with +/- was marked the case when a gene had similar expression changes in 2 out of the 3 independent experiments; * HT29 cells were investigated only in 2 independent experiments; ** Down-regulated genes.

| **Pathway** | **Genes** | **Cells** | | | |
| --- | --- | --- | --- | --- | --- |
|  |  | ***HCT116*** | ***HT29**** | ***DU145*** | ***BJ*** |
| **Cell death** | ATG7 | - | - | + | - |
|  | GRB2 | + | - | - | - |
|  | RIPK1 | +/- | + | +/- | - |
|  | TNFRSF10A | + | - | - | - |
|  | TNFRSF10B | + | - | - | - |
|  | TNFRSF1A | +/- | + | + | - |
|  | ULK1 | + | - | + | - |
| **DNA damage& repair** | ATM | - | - | + | - |
|  | CDKN1A | + | + | +/- | +/- |
|  | CHEK2 | - | - | + | - |
|  | DDB2 | + | - | - | - |
|  | HUS1 | +/- | - | - | +** |
|  | RAD17 | +/- | - | + | - |
|  | RAD51 | - | - | - | +** |
|  | RAD9A | + | - | - | - |
| **Unfolded protein response** | ATF6 | - | - | - | +** |
|  | BBC3 | +/- | - | - | - |
|  | CALR | - | + | - | - |
|  | DNAJC3 | +/- | - | - | - |
|  | HSPA4 | +/- | - | - | - |
| **Hypoxic stress** | CA9 | - | + | - | - |
|  | HMOX1 | + | + | +/- | - |
|  | LDHA | +** | - | +/- | - |
|  | MMP9 | + | + | - | - |
|  | SERPINE1 | + | + | - | - |
|  | SLC2A1 | - | + | +/- | - |
|  | VEGFA | + | - | - | - |
| **Antioxidant response** | GCLC | +/- |  | + |  |
|  | GCLM | +/- | + | +/- | - |
|  | GSR | - | - | + | - |
|  | HMOX1 | + | + | - | - |
|  | NQO1 | +** | - | + | - |
|  | PRDX1 | +/- | - | +/- | - |
| **Osmotic stress** | AKR1B1 | + | +/- | + | - |
|  | EDN1 | + | - | - | - |
|  | HSPA4L | + | - | + | - |
|  | NFAT5 | + | - | - | - |
|  | SLC5A3 | + | + | + | + |
